# Integrating COX-2, stromal PD-L1, and T-cell infiltration enhances prognostic stratification in colorectal cancer

**DOI:** 10.1186/s12885-025-14927-x

**Published:** 2025-09-16

**Authors:** Geriolda Topi, Anita Sjölander, Shakti Ranjan Satapathy

**Affiliations:** 1 Cell and Experimental Pathology, Department of Translational Medicine, Lund University, Skåne University Hospital, Jan Waldenströms gata 35, Malmö, 205 02 Sweden; 2 Department of Endocrinology, Skåne University Hospital, Malmö, Sweden

**Keywords:** COX-2, 15-PGDH, PD-L1, Immune cells, Colon cancer

## Abstract

**Background:**

Colorectal cancer (CRC) is shaped by a complex tumor immune microenvironment in which inflammatory mediators like cyclooxygenase-2 (COX-2) and immune checkpoints such as programmed cell death ligand 1 (PD-L1) play central regulatory roles. However, the prognostic significance of these makers, individually and in combination with T-cell infiltration, remains poorly defined.

**Methods:**

We investigated the interplay between PD-L1, COX-2, 15-hydroxy prostaglandin dehydrogenase (15-PGDH), and TILs using both an internal immunohistochemistry (IHC)-based CRC cohort (*n* = 320) and large public transcriptomic datasets (GSE39582, TCGA-COAD, and E-MTAB-12862). Immune cell composition was analyzed using CIBERSORTx and single-sample gene set enrichment analysis (ssGSEA), and tumors were stratified by consensus molecular subtypes (CMS). Prognostic relevance was evaluated through survival modeling and risk signature development.

**Results:**

PD-L1 expression in the tumor stroma (TS⁺ PD-L1) demonstrated stronger prognostic value than cancer cell-specific PD-L1 (CC^+^ PD-L1) and correlated positively with COX-2 expression and TIL abundance. Across all cohorts, *CD274*, *PTGS2*, and *CD8A* expression were strongly correlated and enriched in CMS1 tumors. High *PTGS2* tumors exhibited inflamed but often immunosuppressive phenotypes, marked by elevated IFN-γ and inflammatory response pathway scores. Multivariate survival analyses confirmed that the combined expression of TS⁺ PD-L1, COX-2, and TIL markers outperformed single-marker models. A composite IHC-based immune-inflammation risk score improved prognostic prediction over TNM staging alone.

**Conclusion:**

PD-L1 and COX-2 define a conserved immunoregulatory axis in CRC that shapes tumor-immune interactions and impacts prognosis. Integration of TS^+^ PD-L1, inflammatory markers, and T-cell contexture enhances risk stratification and may inform future immunotherapeutic and chemopreventive strategies.

**Supplementary Information:**

The online version contains supplementary material available at 10.1186/s12885-025-14927-x.

## Introduction

Chronic inflammation, sustained by an imbalance of pro- and anti-inflammatory molecules, including lipid mediators, supports the initiation and progression of colorectal cancer (CRC)-the third most commonly diagnosed and second deadliest malignancy worldwide [1–3]. Among the implicated lipid mediators, arachidonic acid (AA) and its metabolites have been identified as key regulators of tumor-promoting inflammation. AA metabolizes into either ‘prostanoids’ (prostaglandins and thromboxane) via the Cyclooxygenase (COX) pathway or into ‘leukotrienes’ (LTs) via the lipoxygenase (LOX) pathway [4].

PGE_2_ binds to the high-affinity G-protein-coupled receptors EP2 or EP4, activating multiple oncogenic pathways to initiate and sustain CRC tumors. Furthermore, *PTGS2* protein (COX-2, HUGO Gene Nomenclature Committee (HGNC): 9605) has been well studied as a mediator of CRC, with high expression noted in approximately 50% of adenomas and 85% of adenocarcinomas [4, 5] and is well correlated with poor patient prognosis [6, 7]. Over the past few years, extensive research on the COX-2/PGE_2_/EP4 signaling axis has led to the development of targeted therapies, such as COX inhibitors and EP2/4 inhibitors [8–10]. Additionally, the potential therapeutic benefit of non-steroidal anti-inflammatory drugs (NSAIDs) like *Aspirin* has been explored in large cohort studies on CRC patients, including the ALASCCA trial study, yielding notable outcomes [11–14]. Moreover, long-term *Aspirin* use has demonstrated remarkable efficacy in CRC prevention and is associated with reduced COX-2 expression, underscoring the importance of inflammatory mediators in CRC tumorigenesis [15]. In a recent study, Yang et al. have uncovered the underlying mechanism of *Aspirin* in preventing metastasis in CRC [16].

In normal colonic epithelium, PGE_2_ is rapidly inactivated by the NAD^+^-dependent oxidase 15-hydroxy prostaglandin dehydrogenase (15-PGDH), which catalyzes its conversion into the inactive metabolite 15-keto PGE_2_ [17]. However, *HPGD* (15-PGDH, HGNC: 5154) protein is significantly downregulated in colorectal tumors, and genetic ablation of this enzyme promotes colitis and polyp formation in mouse models [18]. We have also previously reported that patients with high expression of 15-PGDH in colon tumor tissue have better prognoses [19, 20]. Additionally, 15-PGDH in CC cells is activated via either WNT5A activation or cysteinyl leukotriene receptor 2 (CysLT_2_R) activation, which promotes differentiation and reduces proliferation [19, 20]. Furthermore, CysLT_2_R-mediated upregulation of 15-PGDH in CC cells is regulated via hedgehog/GLI signaling [21].

Immune checkpoint blockade (ICB) therapies targeting *CD274* (programmed death ligand-1; HGNC:17635) protein or *PDCD1* (Programmed death 1, HGNC: 8760) protein have demonstrated significant clinical success in treating solid tumors such as melanoma and lung carcinoma [22–25]. However, in CRC, clinical responses to anti-PD-1 or anti-PD-L1 antibodies are restricted primarily to tumors with mismatch repair deficiency (dMMR) or high microsatellite instability (MSI-H), which represent only a small subset of cases [26, 27]. This highlights an urgent need to identify biomarkers predictive of ICB efficacy and disease prognosis in the broader CRC population. Although tumor-infiltrating lymphocytes (TILs) are key mediators of anti-tumor immunity, their prognostic value in CRC remains context-dependent, and the role of *CD274* (PD-L1) protein in modulating TIL activity in this setting remains controversial [28–32]. Recently, Cecil et al. reported decreased *CD274* (PD-L1) protein expression and increased TILs in CC with *PTGS2* (COX-2) protein inhibitors [33]. However, the correlation between the expression of *PTGS2* (COX-2) protein and *CD274* (PD-L1) protein, or TILs, in CRC patients and their significance for patient survival remains unexplored.

Here, we investigate the prognostic significance of *CD274* (PD-L1) protein expression in CRC and its relationship with *PTGS2* (COX-2) protein, *HPGD* (15-PGDH) protein, and immune infiltration. By integrating molecular, immunological, and clinicopathological parameters, we identify combinatorial biomarker signatures that stratify patient outcomes and illuminate novel therapeutic vulnerabilities in the inflammatory tumor microenvironment (TME).

## Materials and methods

### Data acquisition and preprocessing (Public datasets)

#### GSE39582 expression and clinical data processing

We used the microarray-based dataset GSE39582 [34], comprising CRC samples profiled using Affymetrix Human Genome U133 Plus 2.0 Array (GPL570). Expression and clinical metadata were retrieved from the GEO platform (GEO Accession viewer) using the *GEOquery* package (v2.66.0) in R (v4.2.0). Expression values were quantile-normalized and log2-transformed using the *preprocessCore* package (v1.62.1). Probe-level identifiers were converted to gene symbols based on the platform annotation file, and multiple probes mapping to the same gene were averaged. Clinical annotations, including overall survival (OS) data, were extracted from the *pData* slot of the ExpressionSet. Samples with missing expression or survival data were excluded. The final processed dataset was used for immune deconvolution, gene expression correlations, consensus molecular subtype (CMS) classification, and survival analysis.

#### E-MTAB-12862 RNA-seq data processing

We accessed the E-MTAB-12,862 dataset from the ArrayExpress repository, which contains RNA-seq data for 1,183 samples [35]. The normalized expression matrix was downloaded and filtered to retain only tumor samples (*n* = 1,063). Expression values were log2-transformed as log2[TPM + 1]. Gene symbols were assigned to row names, with samples as columns. This processed matrix was used for downstream immune and pathway analysis, including CIBERSORTx deconvolution, CMS classification, and single-sample gene set enrichment analysis (ssGSEA).

#### TCGA-COAD RNA-seq data processing

TCGA-COAD expression and clinical data were retrieved from the Genomic Data Commons (GDC) using the *TCGAbiolinks* package. Raw counts for primary tumor samples were obtained. Ensembl gene IDs were mapped to HGNC symbols using the *org.Hs.eg.db*, and version suffixes were removed. Data were normalized to counts per million (CPM) using the *edgeR* package and log2-transformed. The final matrix was used for immune profiling (CIBERSORTx), CMS classification, and ssGSEA.

### Immune cell Deconvolution (CIBERSORTx)

The CIBERSORTx (https://cibersortx.stanford.edu/) platform was used to estimate relative proportions of 22 immune cell subsets based on the LM22 signature [36]. For GSE39582, quantile normalization was applied (the default for microarray data). For TCGA-COAD and E-MTAB-12,862, quantile normalization was disabled as recommended for RNA-seq. Only samples with a deconvolution p-value of less than 0.05 were considered for downstream correlation and clustering analyses.

### Immune cell interactions (TIMER2.0)

To assess tumor-immune interactions, we also referenced the correlation plots from the TIMER2.0 platform [37], focusing on *CD274* and *PTGS2* expression in relation to CD4^+^ and CD8^+^ T-cell infiltration levels.

### Gene set enrichment analysis (GSEA)

Differential gene expression was assessed using the *limma* package [38], comparing *PTGS2*-high vs. *PTGS2*-low samples (median split) separately in TCGA-COAD and E-MTAB-12862. Genes were ranked by moderated t-statistics, and enrichment analysis was performed using the *clusterProfiler* package [39], with Hallmark gene sets from *msigdbr* [40]. False discovery rate (FDR) < 0.05 was considered significant. Key immune and inflammatory pathways were visualized using *dotplot()* and *gseaplot2()*.

### SsGSEA

The ssGSEA was performed using the *GenePattern* web module with Hallmark gene sets (*MSigDB v7.5.1*, category H) [41]. The log-transformed matrices from TCGA-COAD and E-MTAB-12862 were converted to GCT format, and ssGSEA scores were calculated using default parameters. Output scores were visualized and correlated with the expression of *CD274*, *PTGS2*, *CD8A*, and *HPGD* in R using *ggplot2*.

### CMS classification

CMS classification was carried out using the *CMScaller* package (v2.0.1) [42]. For TCGA-COAD and E-MTAB-12,862, normalized gene expression data were input with RNAseq = TRUE. Samples were assigned to CMS1-CMS4 using nearest template prediction (NTP), with an FDR < 0.05 threshold.

### Single-Cell RNA-Seq annotation (TISCH2)

To validate immune markers at the single-cell level, CRC datasets from the TISCH2 portal (http://tisch.comp-genomics.org/) were reviewed [43]. Uniform manifold approximation and projections (UMAPs) were used to visualize the expression of *CD3D*, *CD4*, and *CD8A* across T-cell subsets, including cytotoxic, regulatory (Tregs), and exhausted T-cells in annotated clusters.

### Patient cohorts

Patients included in the study belong to the previously described cohort, the *Female cohort* (internal cohort) [21, 44–47]. The study was approved by the Ethical Committee of Lund University, Sweden (Dnr 3/2006). All participants provided written informed consent for the scientific use of their clinical data and samples. The desired endpoint was OS, with a follow-up time of up to ten years [47]. The follow-up period started on the date of diagnosis and ended on the date of the indicated event or censoring (August 31, 2016). The study complied with the 1975 Declaration of Helsinki, as revised in 1983. The study flowchart for the internal patient cohort is included in the figure.

### Cell line culture and maintenance

Human colon cancer (CC) cells RKO and SW480 were obtained from the American Type Culture Collection (ATCC; NJ, USA) and cultured under standard conditions. RKO and SW480 cells were cultured using MEM (HyClone™, Cytiva Life Sciences, Germany) and RPMI-1640 medium (HyClone™, Cytiva Life Sciences, Germany), respectively, supplemented with 10% fetal bovine serum (FBS; HyClone™, Cytiva Life Sciences, Germany), 1% L-glutamine, and 100 µg/mL penicillin-streptomycin solution at 37 °C in a humidified incubator with 5% CO_2_. MEM medium was also supplemented with 1% non-essential amino acid (Cytiva Life Sciences, Germany). Cells were routinely passaged at 70–80% confluence and regularly tested for mycoplasma contamination.

### Antibodies and reagents

All the antibodies used in the study are Listed in Supplementary Table 1.

### IHC analysis

IHC staining was performed as previously described [21, 47]. Antibody dilutions and corresponding details are provided in Supplementary Table 1. Antigen retrieval was carried out using DAKO retrieval solutions (pH 6.0 or 9.0) (S236984-2, DAKO, Denmark), selected according to the manufacturer’s recommendations for each antibody. All staining procedures were conducted using the Leica Histocore Spectra ST automated stainer (Leica Biosystems, Sweden) at the Tissue Microarray Core Facility, Department of Translational Medicine, Lund University, Malmö, Sweden.

Halo imaging software (HALO version 3.0, Indica Labs, New Mexico, USA) was used to quantify the expression of membrane PD-L1 (cancer cell positive, CC^+^) (Membrane module v1.4), COX-2 (Cytonuclear module v1.3), and 15-PGDH (Cytonuclear module v1.3) based on the digital H-score. Meanwhile, tumor stroma positive (TS^+^) PD-L1 and immune cells (CD3^+^, CD4^+^, and CD8^+^) were calculated as the number of positive cells (Immune cell module v1.3).

### MSI status evaluation in patients

MSI status was determined by IHC staining of MMR proteins (MLH1, MSH2, MSH6, PMS2). Patients with preserved nuclear staining for all four markers in the tumor cells were considered as MMR-proficient (pMMR) or microsatellite stable (MSS), and patients with loss of nuclear staining in either of the four markers were categorized as dMMR or MSI.

### Composite risk score calculation

To quantify the combined prognostic effect of *CD274* (PD-L1), *PTGS2* (COX-2), and *CD8A*/TIL expression, we constructed a composite risk score based on a multivariate Cox proportional hazards model. Expression data from GSE39582 or the internal cohort were entered into the model, and the corresponding regression coefficients (β) were extracted for each marker. The risk score for each patient was calculated as a weighted linear combination of expression levels of the marker, with the weights corresponding to the estimated Cox regression coefficients:

Risk Score_i = (β₁ × CD274_i) + (β₂ × PTGS2_i) + (β₃ × CD8A_i)

CD274_i, PTGS2_i, and CD8A_i represent the gene/protein expression values for patient i (where i is an individual patient), and β₁, β₂, and β₃ are the Cox regression coefficients reflecting the strength and direction of association with OS.

The resulting composite risk score represents the patient’s relative hazard and was used to stratify the cohort into high- and low-risk groups using the median risk score as a cutoff. Kaplan-Meier survival analysis and time-dependent ROC curves were then used to evaluate the prognostic performance of the score. This approach allows for the integration of multiple biomarkers into a single prognostic index, improving interpretability and enabling risk-based stratification in clinical settings.

### Immunofluorescence analysis

RKO and SW480 CC cells (5 × 10^4^ cells per well) were cultured on coverslips and treated with IFN-γ (50 ng/mL) for 24 h to induce PD-L1 expression, followed by treatment with the COX-2 inhibitor *Celecoxib* (10 µM) for an additional 24 h. Cells were fixed with 4% paraformaldehyde (Thermo Fisher Scientific, Germany) for 10 min, permeabilized using 0.1% Triton X-100 for 15 min, and blocked with 5% BSA for 1 h at room temperature. Dual immunofluorescence staining was performed using primary antibodies against COX-2 and PD-L1, followed by species-specific Alexa Fluor-conjugated secondary antibodies (Anti-rabbit Alexa Fluor 488 and anti-mouse Alexa Fluor 555). PD-L1 is presented as green and COX-2 is presented as pseudo color (magenta) for better visualization. Nuclei were counterstained with DAPI. Imaging was performed using Z-stack in a Leica LSM700 confocal microscope and projected with maximum intensity. The fluorescence intensity was analysed using macros in ImageJ (NIH, USA). Representative images from at least three independent experiments are presented.

### Statistics

Statistical analyses were performed using GraphPad Prism (version 10.0, GraphPad Software, Inc., San Diego, CA, USA), SPSS version 23.0 (SPSS, IBM, Armonk, NY, USA), and R (version 4.2.0), unless stated otherwise. All statistical tests were two-sided, and a *p*-value < 0.05 was considered statistically significant unless otherwise specified.

Survival and prognostic analysis: Receiver operating characteristic (ROC) curve analysis was used to evaluate the predictive performance of individual genes and combined markers. The Youden index (*J = Sensitivity + Specificity –* 1) was applied to determine the optimal cutoff thresholds for stratifying high- vs. low-risk patients.

Univariate and multivariate Cox proportional hazards regression models were employed to assess the association between gene expression and OS. HRs and corresponding 95% confidence intervals (CIs) were reported. Multivariate models included covariates that were significant in univariate analysis and were adjusted for potential confounders: age, sex, and TNM stage in the GSE39582 cohort; age and TNM stage in the internal Female cohort. Patients with incomplete expression or clinical data were excluded from the respective analyses. Clinico-pathological covariates in the low or high TS^+^ PD-L1 patients’ groups are listed in Table 1. Clinical variables used in modeling are summarized in Table 2 (Female cohort) and Supplementary Table 1 (GSE39582).

**Table 1:** Distribution of clinical and pathological covariates in colorectal cancer patients according to tumor stroma (TS^+^) PD-L1 expression

Characteristic	Total *N* (%)		High TS^+^ PD-L1 *N* (%)		Low TS^+^ PD-L1 *N* (%)		*P*
Patients no.	193	100.0	85	45.0	107	55.0	
Age (mean, years)	71.3		70.9		71.6		0.002^b^
Death	54	100.0	26	48.1	28	51.9	
CRC localization							
Colon Rectum	170 23	88.1 11.9	75 10	88.5 11.5	94 13	87.7 12.3	0.870^a^
TNM stage							
I II III IV Missing	33 80 61 17 2	17.3 41.9 31.9 8.9	5 38 30 13	5.8 44.2 34.9 15.1	28 42 31 4	26.7 40.0 29.5 3.8	< 0.001^a^
Tumor extent							
T1 T2 T3 T4 Missing	20 22 124 26 1	10.4 11.5 64.6 13.5	3 6 62 16	3.4 6.9 71.3 18.4	17 16 62 10	16.2 15.2 59.0 9.5	0.003^a^
Nodal status							
N0 N1 N2 Missing	119 46 27 1	62.0 24.0 14.1	48 27 12	55.2 31.0 13.8	71 19 15	67.6 18.1 14.3	0.104^a^
Distant metastasis							
M0 M1	176 17	91.2 8.8	74 13	85.1 14.9	102 4	96.2 3.8	0.006^a^
Treatment after operation							
No treatment Adjuvant treatment Palliative treatment Missing	126 57 5 5	67.0 30.3 2.7	53 28 4	62.4 32.9 4.7	73 29 1	70.9 28.2 1.1	0.192^a^
MMR status							
MSI (dMMR) MSS (pMMR)	103 90	53.4 46.6	31 56	35.7 64.3	72 34	67.9 32.1	< 0.001^c^

^a^Pearson chi-square test

^b^Fisher’s Exact Test

**Table 2:** Survival and associations with clinicopathologic factors in the internal cohort using Cox regression

Clinicopathologic variables	Internal cohort
	Overall Survival HR 95%CI *p*-value
Age	
≥ 65 vs. ˂65	3.130 1.776–5.518 <0.001
TNM Stage	
All stages	1.627 0.663–3.996 <0.001
CC^+^ PD-L1 expression	
Categorical (High vs. Low)	1.916 0.966–3.797 0.063
TS^+^ PD-L1 expression	
Categorical (High vs. Low)	1.644 0.961–2.812 0.07
COX-2 expression	
Categorical (High vs. Low)	1.851 1.046–3.273 0.034
15-PGDH expression	
Categorical (High vs. Low)	0.695 0.398–1.216 0.202
CD3 expression	
Categorical (High vs. Low)	0.360 0.143–0.907 0.030
CD4 expression	
Categorical (High vs. Low)	0.391 0.204–0.748 0.005
CD8 expression	
Categorical (High vs. Low)	0.584 0.335–1.019 0.058

Correlation and group comparisons: Spearman’s rank correlation was used to evaluate associations between continuous variables. Group-wise comparisons were performed using the Wilcoxon rank-sum test or the Kruskal-Wallis test, depending on the number of groups being compared.

Visualization and software: Data visualization, including scatterplots, boxplots, heatmaps, and UMAPs, was performed using the R packages *ggplot2*, *patchwork*, and *pheatmap*. All figures include annotations of *p*-values or correlation statistics as appropriate.

## Results

### A conserved PTGS2-CD274-CD8A axis defines immune interactions across CRC datasets

To determine whether prostaglandin signaling and immune checkpoint activation are functionally linked in CRC, we first assessed the correlation between *PTGS2*, *CD274*, and *CD8A* expression across three independent CRC transcriptomics cohorts: GSE39582, TCGA-COAD, and E-MTAB-12,862.

**Fig. 1:**
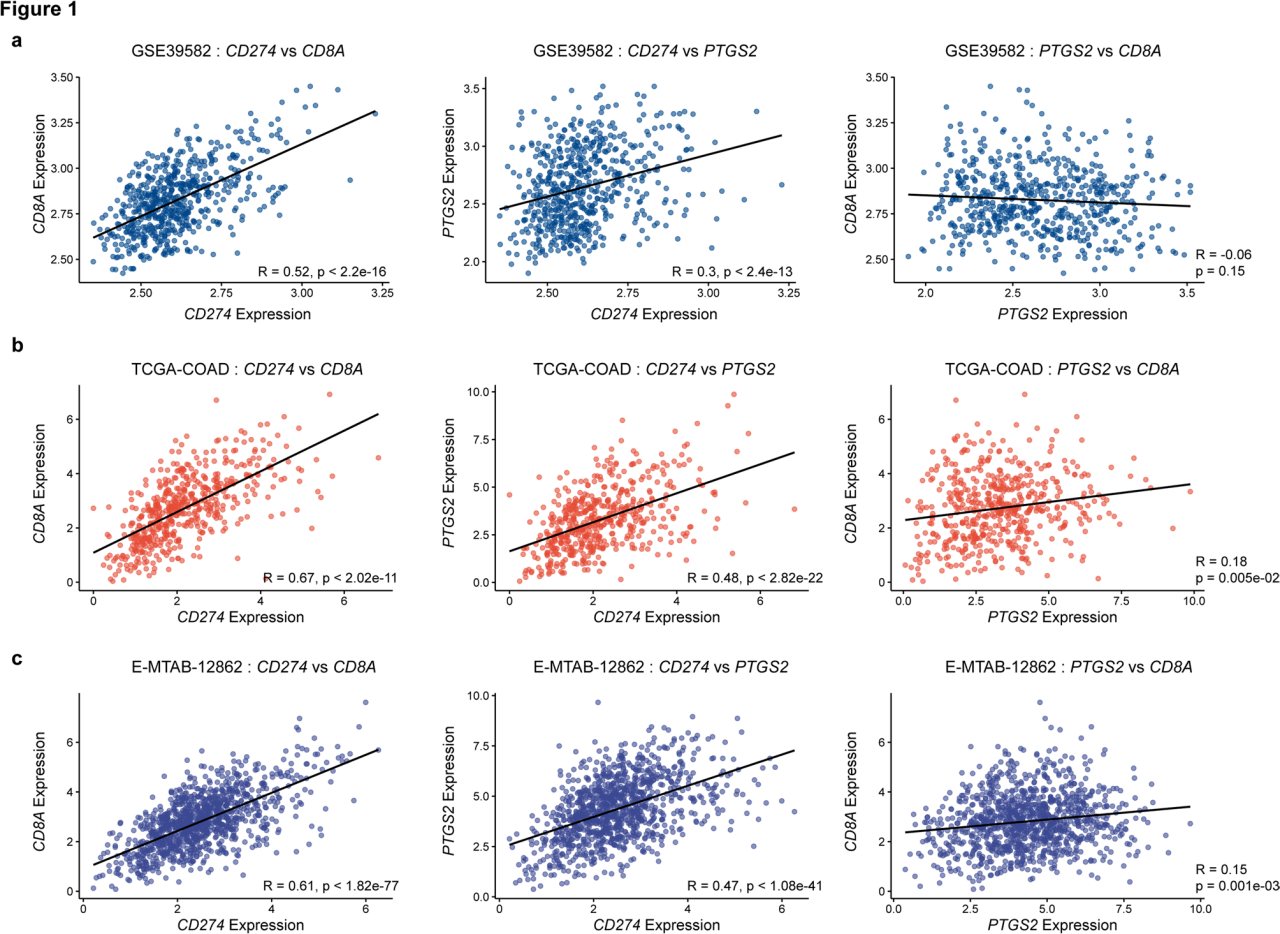
Fig. 1. A conserved PTGS2-CD274-CD8A axis defines immune interactions across CRC datasets.Scatterplots showing pairwise Spearman correlations between CD274 (PD-L1), PTGS2 (COX-2), and CD8A (CD8) expression in the **a** GSE39582 cohort, **b** TCGA-COAD cohort, and **c** E-MTAB-12862 cohort. Each point represents one patient sample, and regression lines were fitted using linear modeling. CD8A is used as a surrogate marker for the presence of cytotoxic T cells. CD274 and PTGS2 are consistently co-expressed (GSE39682, R = 0.3; TCGA-COAD, R = 0.48; E-MTAB-12862, R = 0.47) and correlate with CD8A (GSE39682, R = 0.52; TCGA-COAD, R = 0.67; E-MTAB-12862, R = 0.61) across all three cohorts. The Spearman correlation coefficient is represented by ‘R’. P-value < 0.05 was considered statistically significant.PD-L1; Programmed cell death Ligand 1, COX-2; Cyclooxygenase 2, CD; Cluster of differentiation

In the GSE39582 dataset, weak to moderate positive correlations were observed between *CD274* and *PTGS2* (*R* = 0.3) and between *CD8A* and *CD274* (*R* = 0.52) (Fig. 1a). These correlations were moderate to strong as observed in the TCGA-COAD RNA-seq cohort (*CD274* vs. *PTGS2*, *R* = 0.48; *CD8A* vs. *CD274*, *R* = 0.67) (Fig. 1b) and similar when validated in the E-MTAB-12,862 cohort, comprising over 1,000 CRC tumor samples (*CD274* vs. *PTGS2*, *R* = 0.47; *CD8A* vs. *CD274*, *R* = 0.61) (Fig. 1c). These consistent patterns suggest a conserved immunoregulatory axis in CRC, wherein COX-2 and PD-L1 are co-expressed and track with cytotoxic T-cell infiltration.

### Immune Deconvolution and pathway enrichment reveal inflamed and suppressive tumor phenotypes

**Fig. 2:**
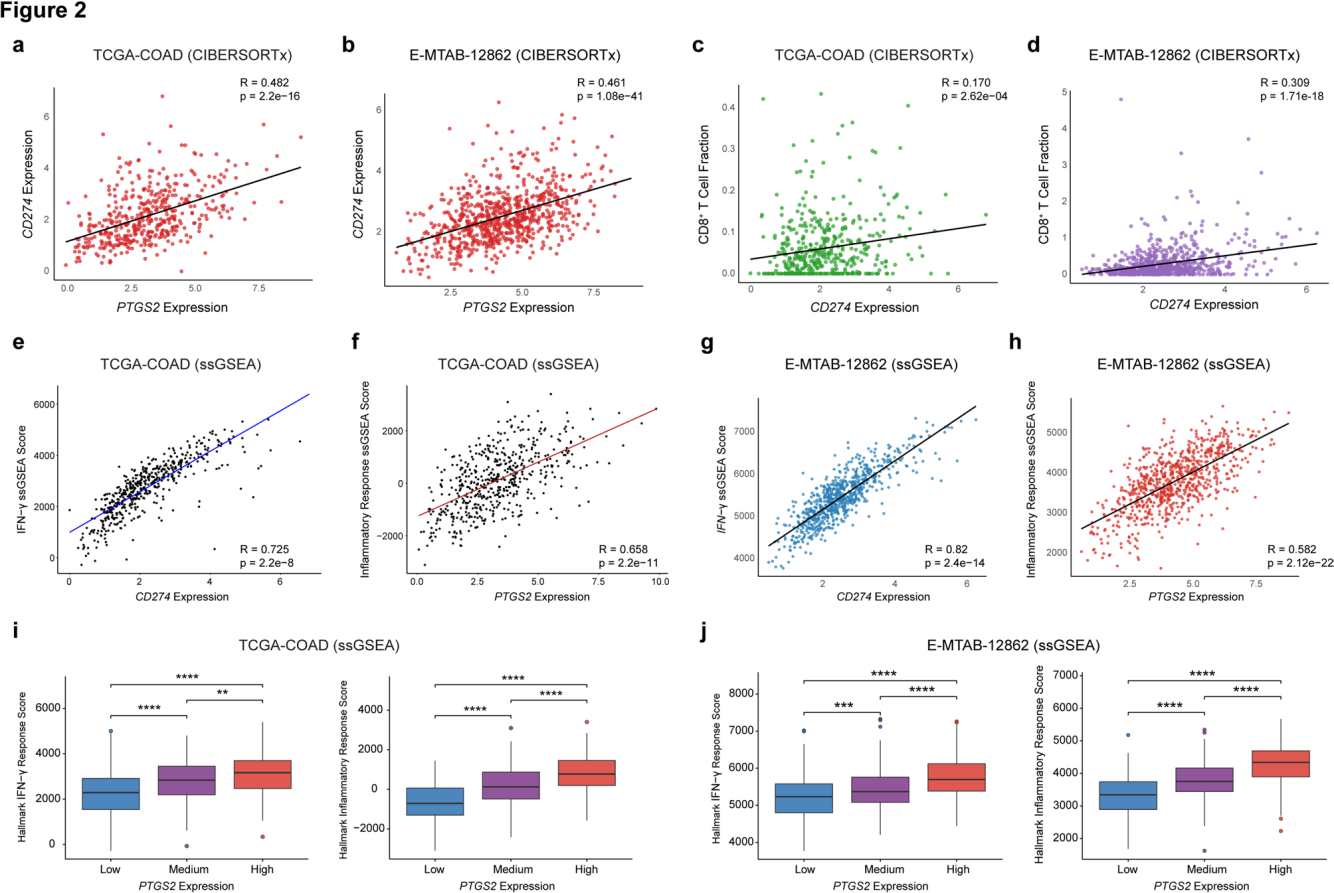
Immune deconvolution and pathway enrichment reveal distinct inflamed and suppressive phenotypes.**a-b** Scatter plots illustrating correlations between *CD274* and *PTGS2* expression in **a** TCGA-COAD and **b** E-MTAB-12,862. **c-d** Scatter plots showing relationships between *CD274* expression and CD8^+^ T-cell infiltration in **c** TCGA-COAD and **d** E-MTAB-12,862. **e-f** Single-sample gene set enrichment analysis (ssGSEA) reveals significant associations between **e** *CD274* expression and IFN-γ pathway activity, and **f** *PTGS2* expression and inflammatory response scores in TCGA-COAD. **g** Box plots stratifying TCGA-COAD patients by *PTGS2* expression tertiles (low/medium/high), demonstrating elevated IFN-γ and inflammatory response scores in high-*PTGS2*-expressing tumors. **h-i**, Replication of ssGSEA correlations in E-MTAB-12,862: **h** *CD274* vs. IFN-γ activity and **i** *PTGS2* vs. inflammatory response. **j** Consistent with TCGA-COAD, E-MTAB-12,862 patients with high *PTGS2* expression exhibit heightened IFN-γ and inflammatory pathway activity. Tumors with elevated *PTGS2* expression demonstrate robust inflammatory and IFN-γ pathway activation in both cohorts, supporting prostaglandin signaling’s role in immune engagement. Spearman correlation coefficient is represented by ‘R’. P-value < 0.05 was considered statistically significant. PD-L1; Programmed cell death Ligand 1, COX-2; Cyclooxygenase 2, CD; Cluster of differentiation

To comprehensively characterize the immune landscape of *PTGS2*- (COX-2) and *CD274* (PD-L1) expressing tumors, we applied CIBERSORTx for immune cell deconvolution and ssGSEA for pathway activity quantification in both the TCGA-COAD and E-MTAB-12,862 cohorts (Fig. 2; Supplementary Fig. 1). We noted a significant and positive correlation between the *CD274* and *PTGS2* mRNA expressions in both the cohorts (TCGA-COAD, *R* = 0.482; E-MTAB-12862, *R* = 0.447) (Fig. 2a, b). Additionally, *CD274* expressions were positively associated with high CD8^+^ T-cell infiltration (Fig. 2c, d). Our analyses also revealed a significant positive correlation between *CD274* expression and IFN-γ pathway activity (TCGA-COAD, *R* = 0.725; E-MTAB-12862, *R* = 0.658), as well as between *PTGS2* expression and inflammatory response signatures in both datasets (TCGA-COAD, *R* = 0.82; E-MTAB-12862, *R* = 0.582) (Fig. 2e-h). These results suggest that COX-2-mediated signaling contributes to shaping an inflammatory TME.

To further dissect this association, we stratified both TCGA-COAD and E-MTAB-12,862 samples by *PTGS2* expression tertiles (low, medium, and high). Notably, patients with high *PTGS2* expression exhibited significant elevations in both IFN-γ and inflammatory response ssGSEA scores compared to those with low or medium expression (Fig. 2i, j). This stratification underscores the dual nature of inflammation in CRC, marked by both immunogenic and suppressive features.

To independently validate these findings, we analyzed TCGA-COAD data using the TIMER 2.0 platform, which estimates immune infiltration from bulk RNA-seq data via constrained deconvolution. Consistent with our prior results, *CD274* expression was positively correlated with both *CD8A* mRNA levels (*R* = 0.672) and estimated CD8^+^ T-cell infiltration (*R* = 0.573) (Supplementary Fig. 2a, b). *CD274* also correlated with *CD4* mRNA expression, although the relationship with CD4^+^ T-cell infiltration was less pronounced. Similarly, *PTGS2* expression showed a positive correlation with both *CD274* mRNA levels (*R* = 0.513) and CD8^+^ T-cell infiltration (*R* = 0.286), reinforcing the link between COX-2 and immune activation (Supplementary Fig. 2c-f).

To further validate these findings at the single-cell level, we analysed three publicly available scRNA-seq datasets of CRC (GSE108989, GSE136394, and GSE146771_10X) accessed via the TISCH2 portal. UMAP projections delineated annotated immune and stromal cell populations, enabling precise visualization of the TME (Supplementary Fig. 3a-c). Feature plots demonstrated that expression of key immune genes, *CD3D* (pan-T cell marker), *CD8A* (cytotoxic CD8⁺ T cells), and *CD4* (helper T cells), was primarily restricted to their expected T cell compartments (Supplementary Fig. 3a’-c’). This cellular specificity confirmed that bona fide immune cell populations drove the immune signatures identified in bulk RNA-seq analyses.

While CIBERSORTx identified a spectrum of immune subtypes, TIMER 2.0 independently confirmed the central CD8^+^ T-cell-PD-L1 axis, providing orthogonal validation of immune involvement in *PTGS2*-high tumors. Together, these findings demonstrate that the inflammatory and immunosuppressive signatures detected in our multi-cohort analyses are robust across both expression-based and infiltration-based estimation methods, highlighting the complex interplay between COX-2, PD-L1, and the immune microenvironment in CRC.

### CMS contextualizes inflammation and immune activation

**Fig. 3:**
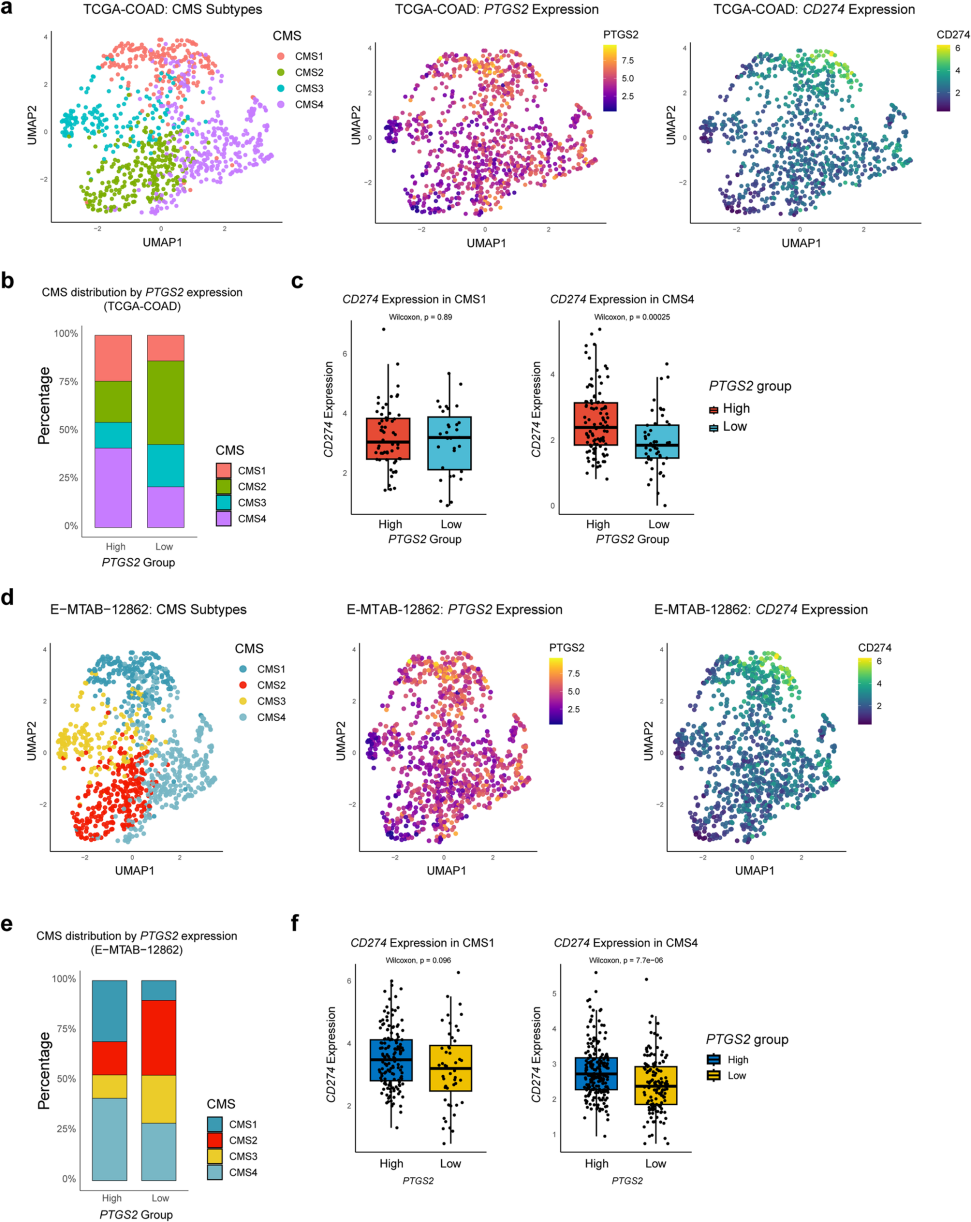
CMS subtypes contextualize immune and inflammatory expression patterns. **a** UMAPs showing the consensus molecular subtypes (CMS) 1–4, *PTGS2* and *CD274* expressions, and **b** stacked bar plot showing the distribution of CMS subtypes across *PTGS2*-high vs. low tumors in the TCGA-COAD dataset. **c** *CD274* expression in CMS1 and CMS4 subtypes of patients in TCGA-COAD cohort stratified based on *PTGS2* expression (high or low). **d** UMAPs showing the consensus molecular subtypes (CMS) 1–4, *PTGS2* and *CD274* expression, and **e** stacked bar plot showing the distribution of CMS subtypes across *PTGS2*-high vs. low tumors in E-MTAB-12,862 dataset. **f** *CD274* expression in CMS1 and CMS4 subtypes of patients in E-MTAB-12862 cohort stratified based on *PTGS2* expression (high or low). The Wilcoxon test was performed to evaluate statistical significance, and a p-value < 0.05 was considered statistically significant. PD-L1; Programmed cell death Ligand 1, COX-2; Cyclooxygenase 2, CD; Cluster of differentiation

Given the known heterogeneity of CRC biology, we next assessed whether the *PTGS2*-*CD274* axis is associated with specific CMS (Fig. 3; Supplementary Fig. 4). In both the TCGA and E-MTAB cohorts, *PTGS2*-high tumors were significantly enriched in CMS1 (immune-inflamed) and CMS4 (mesenchymal/inflammatory) subtypes, whereas *PTGS2*-low tumors were more frequent in CMS2/CMS3 (Fig. 3a-c; Supplementary Fig. 4a, b). Within CMS1 tumors, *CD274* expression was significantly higher in *PTGS2*-high vs. *PTGS2*-low samples, while this relationship was not seen in CMS4 tumors (Fig. 3d-f), suggesting a CMS-specific interplay between COX-2 and immune checkpoint expression.

### Validation of the inflammatory-immune axis in an internal CRC cohort confirms location-specific PD-L1 expression and prognostic

**Fig. 4:**
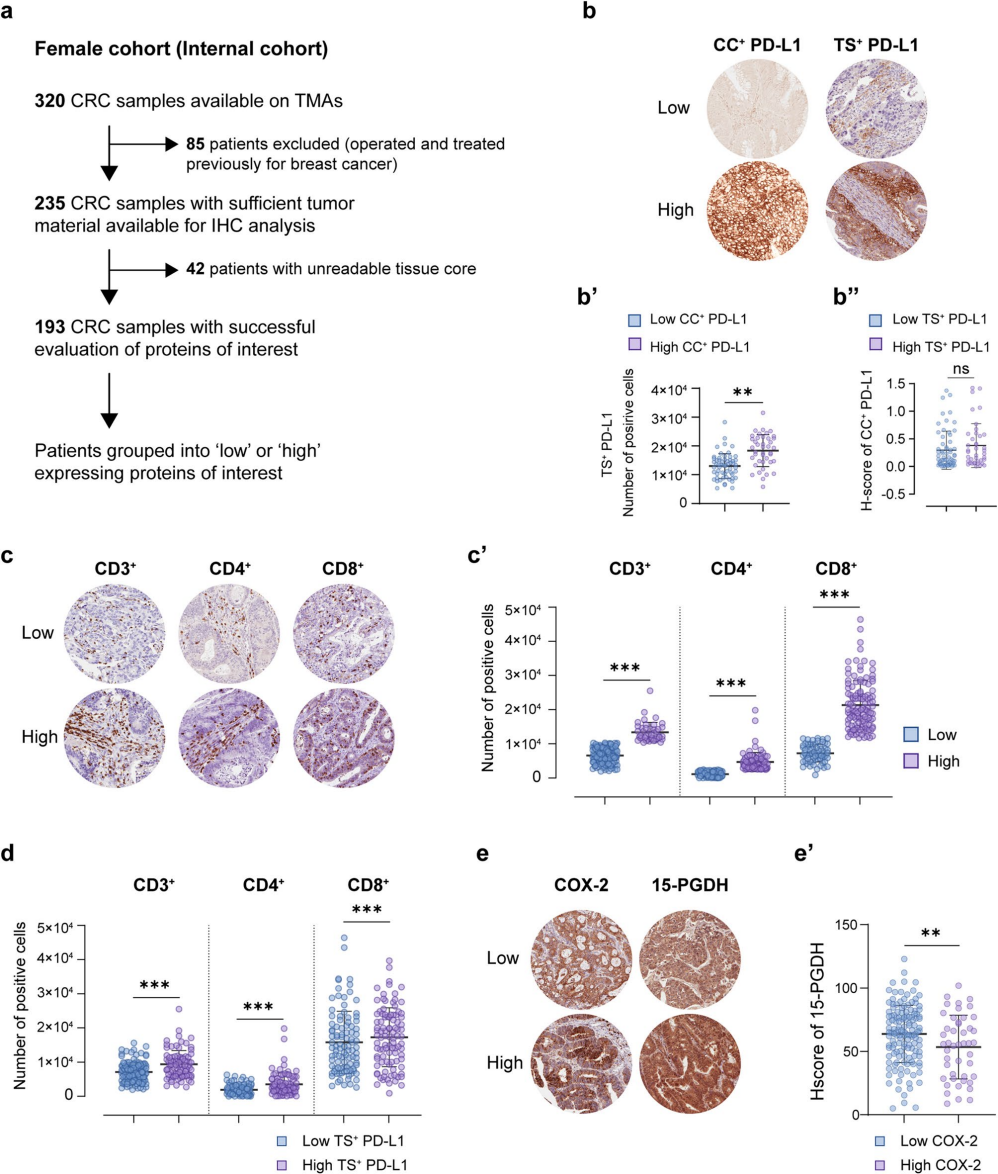
Expression of COX-2, 15-PGDH, PD-L1, and tumor-infiltrating lymphocytes (TILs) in an internal colorectal cancer cohort. **a** Flowchart summarizing the internal colorectal cancer (CRC) patient cohort used for validation. Immunohistochemistry (IHC) analysis was performed on an independent cohort (*f* = 320), of which 193 patients were included in the survival analysis [44–47]. **b** Representative IHC images (20x magnification) showing low and high expression of PD-L1 in CC^+^ or TS^+^, as determined by H-score quantification using Halo Imaging software. **b**’ Dot plots showing differential expression of TS^+^ PD-L1 in patients stratified by CC^+^ PD-L1 levels and **b’’** CC^+^ PD-L1 in patients stratified by TS^+^ PD-L1 levels. **c** IHC images showing representative staining of CD3, CD4, and CD8-positive T-cells. **c’** Dot plots comparing T-cell marker levels in patients classified as high or low for each marker. **d** CD3, CD4, or CD8 expression in patients stratified by TS^+^ PD-L1 expression. **e** IHC images displaying representative high or low expression of COX-2 and 15-PGDH. **e’** Dot plot comparing 15-PGDH H-scores in patients stratified by COX-2 expression status (low, *n* = 138; high, *n* = 46). P values were calculated using Student’s *t*-test. All IHC images were analyzed using the HALO image analysis platform (Indica Labs). PD-L1; Programmed cell death Ligand 1, 15-PGDH; 15-hydroxy prostaglandin dehydrogenase, COX-2; Cyclooxygenase 2, CD; Cluster of differentiation, CC; Cancer cell, TS; Tumor stroma

To evaluate the transcriptomic associations observed in public datasets that also manifest at the protein level, we next analyzed a clinically annotated, IHC-based internal CRC cohort (*n* = 320, female cohort) focusing on PD-L1, COX-2, 15-PGDH, and T-cell markers (CD3, CD4, CD8). Patients were stratified using Youden index-based H-score thresholds and evaluated for spatial PD-L1 expression in cancer cell (CC^+^ PD-L1) and tumor stroma (TS^+^ PD-L1) (Fig. 4a).

We found that TS^+^ PD-L1 and CC^+^ PD-L1 were partially concordant, with high CC^+^ PD-L1 generally aligning with high TS^+^ PD-L1. However, the inverse was not consistently true; many tumors with high TS^+^ PD-L1 lacked CC^+^ PD-L1 expression (Fig. 4b, b’, b’’), suggesting distinct regulatory control in stromal vs. epithelial compartments. Evaluation of T-cell infiltration shows that TS^+^ PD-L1 expression was significantly associated with increased densities of CD3^+^, CD4^+^, and CD8^+^ T-cells, whereas CC^+^ PD-L1 status did not correlate with TIL abundance (Fig. 4c, c’, d; Supplementary Fig. 5). These results position TS^+^ PD-L1 as a more immunologically relevant biomarker than its epithelial counterpart. Moreover, high COX-2 expression was associated with reduced 15-PGDH expression, further supporting the inverse regulation of this prostaglandin metabolic axis at the protein level (Fig. 4e, e’).

In support of these findings, transcriptomic-based survival analysis in the GSE39582 cohort (*n* = 523) revealed that low *CD274* expression was associated with better OS, while high *CD8A* expression predicted a more favorable prognosis (Supplementary Fig. 6a, b). Conversely, high *PTGS2* expression was linked to worse OS (Supplementary Fig. 6c). When stratified into combined expression groups, patients with low *CD274* and high *CD8A* expression had significantly improved survival outcomes (*P* = 0.006), and those with low *CD274* and low *PTGS2* also showed better prognosis (*P* = 0.012) (Supplementary Fig. 6d, e).

### PD-L1, COX-2, and T-cell markers stratify survival outcomes in CRC

**Fig. 5:**
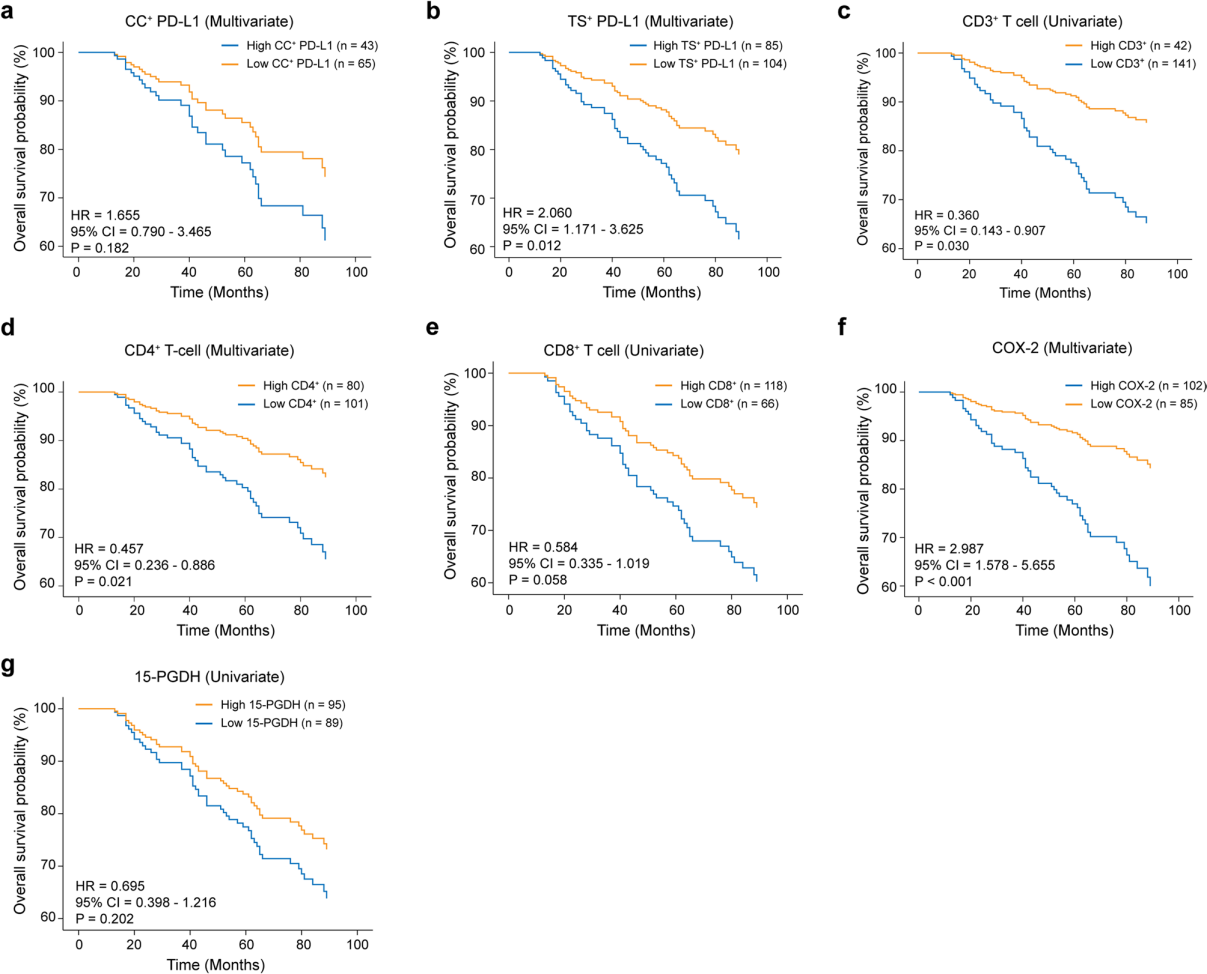
Prognostic value of inflammatory mediators and TILs in an internal colorectal cancer cohort. Kaplan-Meier’s survival analysis was used to visualize overall survival (OS) probabilities in the internal cohort (*n* = 193), with stratification by individual biomarker expression. **a** High vs. low expression of CC^+^ PD-L1; High (*n* = 43) and low (*n* = 65). **b** High vs. low expression of TS^+^ PD-L1; High (*n* = 85) and low (*n* = 104). **c** CD4^+^ T-cell abundance (high, *n* = 80; low, *n* = 101). **d** CD3^+^ T-cell abundance (high, *n* = 42; low, *n* = 141). **e** CD8^+^ T-cell abundance (high, *n* = 118; low, *n* = 66). **f** COX-2 expression (high, *n* = 102; low, *n* = 85). **g** 15-PGDH expression (high, *n* = 95; low = 89). Multivariate survival analysis was performed using Cox proportional hazard modeling, adjusting for clinical variables including age and TNM stage. P values were calculated using the log-rank test. PD-L1; Programmed cell death Ligand 1, COX-2; Cyclooxygenase 2, CD; Cluster of differentiation, CC; Cancer cell, TS; Tumor stroma

To validate the above observed transcriptomics-based survival trend with corresponding protein expressions, we next investigated the individual prognostic value of these markers in the internal cohort using multivariate Cox regression models adjusted for age and TNM stage. Consistent with findings from the GSE39582 dataset, high TS^+^ PD-L1 expression was associated with significantly worse OS compared to the low-expression group (HR = 2.06, *P* = 0.012), while CC^+^ PD-L1 showed a weaker, non-significant trend (Fig. 5a, b; Supplementary Fig. 7a, b).

Among T-cell markers, patients with high CD3^+^ T-cell counts showed improved survival outcomes compared to their low-expression counterparts (Univariate, HR = 0.360, *P* = 0.030; multivariate, HR = 0.443, *P* = 0.090) (Fig. 5c, Supplementary Fig. 7c). Moreover, patients stratified into high or low CD4 or CD8 also exhibited similar outcomes, with patients with either high CD4 or high CD8 having a more favorable prognosis than those with low CD4 (Univariate, HR = 0.391, 95% CI = 0.204–0.748, *P* = 0.005; Multivariate, HR = 0.457, 95% CI = 0.236–0.886, *P* = 0.021) (Supplementary Fig. 7 d; Fig. 5d) or low CD8 (Univariate, HR = 0.584, *P* = 0.058; Multivariate, HR = 0.679, *P* = 0.193) counts (Fig. 5e; Supplementary Fig. 7e).

Next, we evaluated the association of *PTGS2* (COX-2) and *HPGD* (15-PGDH) with OS. High *PTGS2* (vs. low) was associated with worse OS in univariate analysis (HR = 1.851; 95% CI, 1.046–3.273; *P* = 0.034) and remained independently prognostic in multivariate models adjusting for age and TNM stage (HR = 2.987; 95% CI, 1.578–5.655; *P* < 0.001) (Fig. 5f; Supplementary Fig. 7f). High *HPGD* (vs. low) showed a trend toward better OS but did not reach statistical significance (univariate HR = 0.695; 95% CI, 0.398–1.216; *P* = 0.202; multivariate HR = 0.889; 95% CI, 0.485–1.630; *P* = 0.703) (Fig. 5g; Supplementary Fig. 7 g).

We also assessed MMR proteins (MLH1, PMS2, MSH2, MSH6) by IHC and classified patients as dMMR/MSI or pMMR/MSS (Supplementary Fig. 8a). In an exploratory subgroup analysis, MSI cases exhibited higher CD3^+^/CD4⁺/CD8⁺ TIL densities and lower TS⁺ PD-L1, compared with the MSS subgroup (Supplementary Fig. 8b, c). In line with these immune associations, MMR status was significantly correlated with TS^+^ PD-L1 distribution (Table 1), with dMMR/MSI cases enriched in the low TS^+^ PD-L1 subgroup (Supplementary Fig. 8b,c). However, when modelled as an independent covariate in Cox regression, MMR status did not show prognostic significance (HR = 0.81, 95% CI: 0.46–1.40, *p* = 0.446). These findings are consistent with the GSE39582 dataset, where MMR status did not stratify OS, suggesting that although MMR deficiency shapes the immune microenvironment, prognosis is better explained by immune contexture than by MMR status itself.

### Combinatorial immune and inflammatory States reveal cooperative survival impact

Given the immune-suppressive potential of prostaglandin signaling, we next test whether PD-L1 associates with COX-2, 15-PGDH, or TIL levels to affect prognosis. To evaluate these hypothetical associations and their impact on OS in the Female cohort, we used the H-score to stratify patients into high TS^+^ PD-L1 and high COX-2 (high TS^+^ PD-L1 + high COX-2) and low TS^+^ PD-L1 and low COX-2 (low TS^+^ PD-L1 + low COX-2) subgroups.

**Fig. 6:**
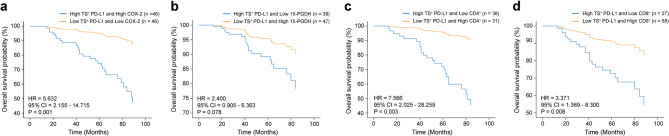
Combined inflammatory and immune features enhance the prognostic impact of tumor stroma-positive PD-L1 expression. Kaplan-Meier’s survival plots showing multivariate overall survival (OS) probabilities in patients stratified by composite biomarker expression states: **a** TS^+^ PD-L1 + COX-2 (high TS^+^ PD-L1 + high COX-2, *n* = 46) vs. low TS^+^ PD-L1 + low COX-2 (*n* = 46). **b **TS^+^ PD-L1 + 15-PGDH (high TS^+^ PD-L1 + low 15-PGDH, *n* = 38) vs. low TS^+^ PD-L1 + high 15-PGDH (*n* = 47). **c** TS^+^ PD-L1 + CD4 (high TS^+^ PD-L1 + low CD4, *n* = 36) vs. low TS^+^ PD-L1 + high CD4 (*n* = 31). **d** TS^+^ PD-L1 + CD8 (high TS^+^ PD-L1 + low CD8, *n* = 27) vs. low TS^+^ PD-L1 + high CD8 (*n* = 58). Multivariate Cox survival analysis included clinical covariates (age and TNM stage). P-values were calculated using the log-rank test. PD-L1; Programmed cell death Ligand 1, 15-PGDH; 15-hydroxy prostaglandin dehydrogenase, COX-2; Cyclooxygenase 2, CD; Cluster of differentiation, TS; Tumor stroma

Patients with high TS^+^ PD-L1 + high COX-2 had significantly worse survival compared to those with low TS^+^ PD-L1 + low COX-2 (HR = 5.632, *P* < 0.001) (Fig. 6a). Similarly, patients with low PD-L1 + high 15-PGDH exhibited better OS than those with high PD-L1 + low 15-PGDH (Fig. 6b). However, the latter comparison did not reach statistical significance (*P* = 0.078). Stratifying patients by TIL abundance alongside TS^+^ PD-L1 revealed that low PD-L1 + high CD4^+^ or CD8^+^ T-cells was associated with improved survival (CD4^+^: HR = 7.57, *P* = 0.003; CD8^+^: HR = 3.37, *P* = 0.008) (Fig. 6c, d). These findings suggest that PD-L1’s prognostic role is shaped by the immune-inflammatory context, particularly by T-cell activity and prostaglandin metabolism.

### A combined inflammatory-immune risk model improves prognostic prediction

**Fig. 7:**
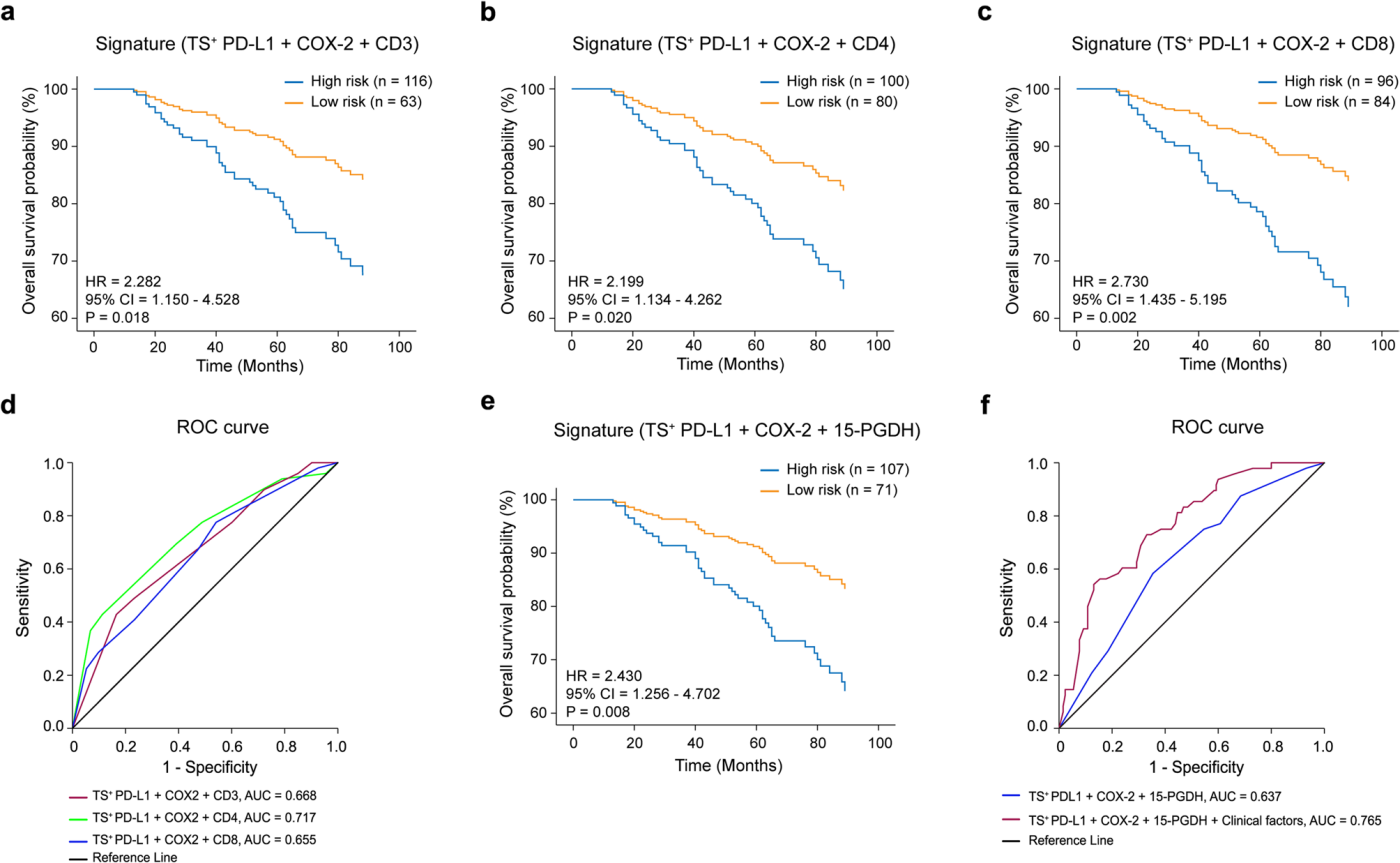
A risk model based on immune and inflammatory markers predicts patient outcome. Kaplan-Meier’s survival plots showing overall survival (OS) probabilities in patients stratified into high- or low-risk groups based on composite biomarker signatures: **a** TS^+^ PD-L1 + COX-2 + CD3 (high-risk, *n* = 116; low-risk, *n* = 63). **b** TS^+^ PD-L1 + COX-2 + CD4 (high-risk, *n* = 100; low-risk, *n* = 80). **c** TS^+^ PD-L1 + COX-2 + CD8 (high-risk, *n* = 96; low-risk, *n* = 84). **d** ROC curves showing AUC for each combined signature: CD3 (AUC = 0.668), CD4 (AUC = 0.717), CD8 (AUC = 0.655). **e** Kaplan-Meier plot for TS^+^ PD-L1 + COX-2 + 15-PGDH (high-risk, *n* = 107; low-risk, *n* = 71). **f** ROC curve comparing AUC of biomarker-only risk score (AUC = 0.637) vs. combined clinical-molecular model (AUC = 0.765), including age and TNM stage. Univariate Cox models were used for survival curves unless otherwise stated. P-values were calculated using the log-rank test. PD-L1; Programmed cell death Ligand 1, 15-PGDH; 15-hydroxy prostaglandin dehydrogenase, COX-2; Cyclooxygenase 2, CD; Cluster of differentiation, TS; Tumor stroma

To assess clinical utility, we developed composite prognostic models incorporating TS^+^ PD-L1, COX-2, and TIL markers (CD3, CD4, and CD8). These combinations were used to stratify patients into high-risk and low-risk groups. The TS^+^ PD-L1 + COX-2 + CD4 model showed the strongest prognostic separation (HR = 2.20, *P* = 0.020) and the highest predictive performance (AUC = 0.717) compared to CD3- or CD8-based models (CD3: AUC = 0.668; CD8: AUC = 0.655) (Fig. 7a-d).

We further evaluated a COX-2 + 15-PGDH + TS^+^ PD-L1 signature, which also stratified risk significantly (HR = 2.43, *P* = 0.008, AUC = 0.637), and its predictive power improved when combined with clinical covariates (AUC = 0.765) (Fig. 7e, f; Supplementary Fig. 9a), supporting the generalizability of the inflammatory-immune risk axis. This observation was in line with results from GSE39582 with *CD274* + *CD8A* or *CD274* + *PTGS2* + *CD8A* have similar AUC values (*CD274* + *CD8A*, AUC = 0.565; *CD274* + *PTGS2* + *CD8A*, AUC = 0.570), which improved when coupled with significant clinical factors (Age, Gender, TNM stages) (Supplementary Fig. 9b, c).

### Celecoxib treatment reduces PD-L1 expression in CRC cells via COX-2 Inhibition

To experimentally validate the association between COX-2 and PD-L1 expression observed in our transcriptomic and IHC analyses, we performed dual immunofluorescence staining in two CC cell lines: RKO (MSI) and SW480 (MSS) (Supplementary Fig. 10). Both cell lines were pre-treated with IFN-γ to induce PD-L1, followed by treatment with the COX-2 inhibitor *Celecoxib*. Confocal microscopy revealed increased PD-L1 expression in IFN-γ-stimulated cells with no significant alteration in the COX-2 expression (Supplementary Fig. 10a, b). However, both PD-L1 and COX-2 expressions in IFN-γ-stimulated cells substantially reduced following *Celecoxib* treatment, supporting a functional relationship between COX-2 activity and immune checkpoint expression in CRC.

## Discussion

Identifying robust prognostic biomarkers remains a clinical priority for enhancing risk stratification in CRC. Over the past two decades, the implications of tumor immune microenvironment-associated prognostic biomarkers have received attention [48]. In CRC, ICB therapy has shown significant success in a context-dependent manner, particularly benefiting patients with MMR deficiency or high MSI [26, 27]. In this study, we comprehensively evaluated the immune-inflammatory axis, comprising PD-L1 (*CD274*), COX-2 (*PTGS2*), 15-PGDH (*HPGD*), and TILs across multiple CRC cohorts and platforms. Our findings uncover a conserved *PTGS2*-*CD274*-*CD8A* axis that defines inflamed yet immune-evasive tumor microenvironments and stratifies prognosis more effectively than single biomarkers.

Building on earlier studies that explored the heterogeneous role of PD-L1 in CRC [28, 32, 49], we began by analyzing gene expression correlations between *PTGS2*, *CD274*, and *CD8A* across three independent datasets: GSE39582, TCGA-COAD, and E-MTAB-12,862. Across all cohorts, we observed consistent, statistically significant positive correlations between these markers, suggesting a conserved transcriptional program that links prostaglandin signaling, immune checkpoint expression, and cytotoxic T-cell infiltration **(**Fig. 1**)**. Immune deconvolution (CIBERSORTx) and pathway enrichment analyses (ssGSEA) further showed that *PTGS2*-high tumors are enriched in CD8^+^ T cells and exhibit elevated inflammatory and IFN-γ pathway activity, reinforcing the biological plausibility of this axis. To contextualize clinical utility, we benchmarked our multi-marker panel (e.g., *PTGS2*-*CD274*-TIL features) against standard clinicopathologic factors in multivariable models. Age and TNM stage were included, and the panel retained independent prognostic value **(**Table 2**)**.

The prognostic role of PD-L1 in CRC remains debated. Some studies associate high PD-L1 expression with favorable outcomes [32, 50, 51], others report no association [28, 52], while several emphasize compartment-specific effect (tumor vs. stromal) [49, 53, 54]. This variability likely reflects methodological differences including, antibody clone, scoring criteria, and biological context. Collectively, these findings suggest that PD-L1 alone has limited prognostic utility, but its value increases when interpreted with immune infiltration and inflammatory mediators. In our analysis, TS^+^ PD-L1 combined with COX-2, 15-PGDH, and TIL density provided the clearest prognostic discrimination.

Our results, showing an interactive association between *PTGS2* (COX-2) and *CD274* (PD-L1) expression in CRC, are consistent with prior epidemiological evidence linking these pathways to patient outcomes. Notably, aspirin improved survival only in PD-L1-negative CRC, implying that immunosuppressive PD-L1 activity may override the benefits of COX-2 inhibition [55]. This is consistent with our observation that COX-2 and PD-L1 are not independent prognostic variables but interact within the same regulatory network. Therapeutically, this highlights the potential of combining COX-2 inhibitors with PD-1/PD-L1 blockade in selected subgroups.

We further examined the role of MMR status. Although MSI/dMMR tumors exhibited higher TIL densities and reduced stromal PD-L1 expression, MMR status alone was not prognostic in either our internal cohort (HR = 0.81, *p* = 0.446) or GSE39582 (HR = 0.943, *p* = 0.917). Instead, immune contexture, particularly high TIL burden and low stromal PD-L1, better explained survival differences. This indicates that the favorable prognosis of MSI/dMMR tumors is mediated by their immune milieu rather than MMR status itself, emphasizing the importance of evaluating immune contexture when assessing prognosis.

From a translational perspective, our panel has practical feasibility: *PTGS2*, *CD274*, *HPGD*, and TILs are assessable by IHC on FFPE tissue, several already in routine use. Importantly, interpretation of PD-L1 IHC must also consider antibody clone variability. Additionally, while our composite score improved patient stratification in survival analysis, its complexity may limit direct adoption in clinical settings, where single-marker or simpler scoring systems are preferred. In clinical pathology, *CD274* (PD-L1) is most commonly reported as the Combined Positive Score (CPS), Tumor Proportion Score (TPS), or immune-cell (IC) score, each based on standardized counting methods and specific diagnostic clones. We used the SP263 clone (Ventana) and assessed PD-L1 positivity as the proportion of tumor and immune cells with membranous staining [56]. Although SP263 has shown high concordance with other clinically validated clones such as 22C3 (PharmDx, Agilent), and 28 − 8 (PharmDx, Agilent) [57], SP142 (Ventana) often reads lower on tumor and immune cells [58]. Consequently, inter-assay and inter-observer variability remain a challenge [56–60], and direct equivalence between scoring systems should be interpreted cautiously. Future studies translating our findings into clinically familiar PD-L1 scoring frameworks and validating across multiple antibody clones are essential to enable clinical implementation.

Importantly, our in vitro data support the biological plausibility of this axis. Treatment with a COX-2 inhibitor reduced PD-L1 expression in both MSI-H and MSS CRC cell lines, reinforcing a potential immunoregulatory role of COX-2 that warrants further therapeutic exploration. Our analyses, based primarily on bulk transcriptomic and tissue-based datasets, are observational and therefore reflect associations rather than direct causal relationships between *PTGS2* (COX-2), *CD274* (PD-L1), and immune cell infiltration. The robustness and reproducibility of these associations across three independent CRC cohorts support their biological relevance. Prior experimental studies have shown that *PTGS2*-mediated prostaglandin E₂ can promote immune evasion by upregulating *CD274* expression and suppressing cytotoxic T-cell and dendritic-cell function [61–63]. In line with these mechanisms, we observed that celecoxib treatment of IFN-γ-stimulated CRC cells (SW480, RKO) reduces *CD274* protein expression (and *PTGS2*) (Supplementary Fig. 10), supporting a functional link between COX-2 signaling and PD-L1 regulation in CRC.

Nonetheless, important limitations remain. The use of TMAs, while efficient, risks underrepresenting intratumoral heterogeneity compared to full-slide or spatial analyses. Our internal validation cohort included only female patients, limiting generalizability to males. Sex-related biology, including hormonal modulation of inflammatory and immune pathways, can influence *PTGS2* (COX-2) and *CD274* (PD-L1) expression and may affect immunotherapy outcomes [64–66]. Although associations in our cohort mirrored those seen in sex-diverse external datasets, validation in gender-balanced cohorts is required to establish robustness and clinical applicability. Moreover, cancer-specific survival (CSS) data were not available in our internal cohort; therefore, OS was used as the primary endpoint. Although CSS can sometimes provide more disease-focused information, it is also prone to misclassification of cause of death, particularly in older patients with comorbidities [67]. By contrast, OS avoids this limitation and enables comparability across cohorts, making it a more robust endpoint in this context.

Beyond standard pathology, our biomarker framework could integrate with artificial intelligence (AI) pipelines [67]. Digital pathology platforms can quantify IHC features and, when combined with molecular and clinical data, enable multimodal prognostic models. Incorporating COX-2, PD-L1, and TIL metrics into such pipelines may facilitate clinical implementation and accelerate adoption into decision-support systems.

In summary, this study identifies a conserved *PTGS2*-*CD274*-*CD8A* axis reproducible across transcriptomic and protein platforms, providing a biologically grounded foundation for prognostic biomarker development in CRC. By integrating stromal PD-L1 with inflammatory mediators and immune infiltration, we demonstrate improved risk stratification over single-marker approaches. Future validation in larger, treatment-annotated, and gender-balanced cohorts, using standardized PD-L1 scoring systems, multiple antibody clones, and spatial profiling methods, will be essential to confirm clinical utility. If validated, this multi-marker panel could refine prognosis, guide immunotherapy strategies, and support integration of anti-inflammatory and checkpoint-targeted therapies in CRC.

## Supplementary Information

Supplementary Material 1.

Supplementary Material 2.

Supplementary Material 3.

Supplementary Material 4.

Supplementary Material 5.

Supplementary Material 6.

Supplementary Material 7.

Supplementary Material 8.

Supplementary Material 9.

Supplementary Material 10.

Supplementary Material 11.

Supplementary Material 12.

Supplementary Material 13.

## Data Availability

The authors confirm that the data supporting this study’s findings are available within the article and/or its supplementary materials. Other relevant data would be shared upon reasonable request to the corresponding author. However, sensitive information regarding the patients included in the study (internal cohort) will not be shared due to privacy concerns. The public dataset GSE39582 can be accessed at [https://www.ncbi.nlm.nih.gov/geo/query/acc.cgi? acc=GSE39582]. The E-MTAB-12862 dataset can be accessed at [https://www.ebi.ac.uk/biostudies/ArrayExpress/studies/E-MTAB-12862].
