# Supplementary material for: Integrating COX-2, stromal PD-L1, and T-cell infiltration enhances prognostic stratification in colorectal cancer
Source: BMC Cancer. 2025 Sep 16;25:1424. doi: 10.1186/s12885-025-14927-x (PMC12442288; doi:10.1186/s12885-025-14927-x)
Supplement: Supplementary file 1 — Supplementary Material 1. [file 12885_2025_14927_MOESM1_ESM.docx]

| Reagents or Antibodies | **Source** | Dilution | **Assay** |
| --- | --- | --- | --- |
| anti-COX-2 (ab52237) | Abcam | 1:100 | Immunohistochemistry |
| anti-COX-2 (35-8200) | Thermo Fisher Scientific | 1:100 | Immunofluorescence |
| anti-15-PGDH (NB200-179) | Novus Biologicals | 1:100 | Immunohistochemistry |
| anti-PD-L1 (SP263) | Roche Diagnostics | Pre-diluted | Immunohistochemistry |
| anti-PD-L1 (13684) | Cell Signalling Technology | 1:100 | Immunofluorescence |
| anti-CD3 (2GV6) | Roche Diagnostics | Pre-diluted | Immunohistochemistry |
| anti-CD4 (SP35) | Roche Diagnostics | Pre-diluted | Immunohistochemistry |
| anti-CD8 (SP57) | Roche Diagnostics | Pre-diluted | Immunohistochemistry |
| Alexa Fluor 488 | Thermo Fisher Scientific | 1:300 | Immunofluorescence |
| Alexa Fluor 555 | Thermo Fisher Scientific | 1:3000 | Immunofluorescence |

**Supplementary Table 1:** List and details of antibodies used in this study.
