# Supplementary material for: Integrating COX-2, stromal PD-L1, and T-cell infiltration enhances prognostic stratification in colorectal cancer
Source: BMC Cancer. 2025 Sep 16;25:1424. doi: 10.1186/s12885-025-14927-x (PMC12442288; doi:10.1186/s12885-025-14927-x)
Supplement: Supplementary file 2 — Supplementary Material 2. [file 12885_2025_14927_MOESM2_ESM.docx]

#### Supplementary file

Integrating COX-2, Stromal PD-L1, and T-Cell Infiltration Enhances Prognostic Stratification in Colorectal Cancer

Geriolda Topi^1,2^, Anita Sjölander^1^, Shakti Ranjan Satapathy^1*^

^1^Cell and Experimental Pathology, Department of Translational Medicine, Lund University, Skåne University Hospital, Malmö, Sweden.

^2^Department of Endocrinology, Skåne University Hospital, Malmö, Sweden.

Keywords: COX-2, 15-PGDH, PD-L1, Immune cells, Colon cancer.

*Address all correspondence to:

Dr. Shakti Ranjan Satapathy, [shakti_ranjan.satapathy@med.lu.se](mailto:shakti_ranjan.satapathy@med.lu.se)

Cell and Experimental Pathology, Department of Translational Medicine,

Clinical Research Center, Lund University, Skåne University Hospital,

Jan Waldenströms gata 35, 205 02 Malmö, Sweden.

Tel: +46-40391168

#### Figure legends

#### Supplementary Fig. 1. Enrichment of Hallmark Pathways in *PTGS2*-High Versus *PTGS2*-Low Tumors Across Two Cohorts

**a, d:** Dot plots showing the top enriched Hallmark pathways in *PTGS2*-high compared to *PTGS2*-low tumors in **a,** TCGA-COAD, and **d,** E-MTAB-12862 cohorts. The x-axis represents the gene ratio (proportion of genes in each pathway that are differentially expressed), while the size of each dot indicates the number of genes (Count), and the color represents the adjusted p-value for enrichment. **b, e:** Gene Set Enrichment Analysis (GSEA) plots for the IFN-γ response pathway in *PTGS2*-high tumors in **b,** TCGA-COAD, and **e,** E-MTAB-12862. The enrichment score (green line) indicates the degree to which the IFN-γ response gene set is overrepresented at the top of the ranked gene list. **c, f:** GSEA plots for the inflammatory response pathway in *PTGS2*-high tumors in **c,** TCGA-COAD, and **f,** E-MTAB-12862. Similar to the above, the enrichment score demonstrates the upregulation of the inflammatory response gene set in *PTGS2*-high tumors.

#### Supplementary Fig. 2. T-cell infiltration correlates with *CD274* and *PTGS2* expression in TCGA-COAD (TIMER 2.0 analysis).

**a,** Scatter plots showing the correlation between *CD274* (PD-L1) and tumor purity (left) or CD8⁺ T-cell infiltration (right). **b,** Scatter plots depicting the correlation between *CD274* and tumor purity (left) or *CD8A* mRNA expression (right). **c,** Scatter plots illustrating the association between *CD274* and tumor purity (left) or CD4⁺ T-cell infiltration (right), as estimated by TIMER2.0. **d,** Scatter plots demonstrating the correlation between *PTGS2* (COX-2) and CD8⁺ or CD4⁺ T-cell infiltration levels. **e,** Positive correlation between *CD274* and *PTGS2* expression in the TCGA-COAD dataset. **f,** Positive correlation between *PTGS2* and CD8^+^ T-cell expression in the TCGA-COAD dataset. All analyses were performed using TIMER2.0 and TCGA-COAD RNA-seq data [1]. Spearman’s correlation coefficient (ρ) and corresponding P-values are indicated.

PD-L1: programmed cell death ligand 1; COX-2: cyclooxygenase-2; CD: cluster of differentiation.

#### Supplementary Fig. 3. Single-cell validation of immune gene expression in colorectal cancer.

UMAP projections from three single-cell RNA sequencing (scRNA-seq) datasets of colorectal cancer (CRC) datasets **a,** GSE108989, **b,** GSE136394, **c,** GSE146771_10X accessed via the TISCH2 portal, showing annotated immune and stromal cell populations. **a’** to **c’,** Feature plots displaying cell-type-specific expression of immune related genes: *CD3D* (pan-T cell marker), *CD8A* (cytotoxic CD8⁺ T cells), and *CD4* (helper T cells) in GSE108989, GSE136394, GSE146771_10X datasets respectively. Gene expression was restricted to expected T cell compartments, validating the cellular specificity of immune signatures identified in bulk RNA-seq analyses.

#### Supplementary Fig. 4. Consensus Molecular Subtype (CMS) Classification Heatmaps in Colorectal Cancer Cohorts.

**a,** CMS classification heatmap for the TCGA-COAD cohort. The heatmap displays the expression of template features (rows) across individual tumor samples (columns) grouped by predicted CMS class (CMS1-CMS4). Red indicates higher expression, and blue indicates lower expression relative to the mean. Distinct expression patterns are observed for each CMS subtype, reflecting characteristic molecular signatures. **b,** CMS classification heatmap for the E-MTAB-12862 cohort. As in **a,** the heatmap shows the expression of template features across samples stratified by CMS class predictions.

#### Supplementary Fig. 5. Cancer cell-specific PD-L1 expression does not associate with T-cell abundance.

Dot plots showing CD3, CD4, and CD8-positive T-cell counts in patients stratified by cancer cell-positive (CC⁺) PD-L1 expression status in the internal CRC cohort. No significant association was observed between CC⁺ PD-L1 and lymphocyte density, indicating limited immune-PD-L1 coupling in tumor epithelial compartments.

#### Supplementary Fig. 6. Combined inflammatory and immune signatures predict prognosis in the GSE39582 cohort.

Kaplan-Meier survival curves showing multivariate overall survival (OS) probabilities in the GSE39582 cohort: **a-c,** Patients stratified by high vs. low expression of *CD274* (n = 116 vs 159), *CD8A* (n = 137 vs. 138), and *PTGS2* (n = 211 vs. 64), respectively. **d-e,** Composite expression models stratified by **d**, *CD274* and *CD8A* or **e**, *CD274* and *PTGS2*, based on Youden index-defined cutoffs. Survival models were adjusted for age, sex, and TNM stage. Log-rank test was used for p-value calculation.

PD-L1; Programmed cell death ligand 1, COX-2; Cyclooxygenase 2, CD; Cluster of differentiation.

#### Supplementary Fig. 7. Prognostic impact of immune and inflammatory markers in the internal CRC cohort.

Kaplan-Meier survival curves evaluating the indicated biomarker expressions: **a,** Univariate OS curves for colon cancer positive (CC^+^) PD-L1, **b,** tumor stroma positive (TS^+^) PD-L1. **c,** Multivariate analysis for CD3^+^ T-cell, **d,** univariate analysis for CD4^+^ T-cell, and **e,** multivariate analysis for CD8^+^ T-cell. **f,** Univariate analysis for COX-2 and **g,** multivariate analysis for 15-PGDH expression. All multivariate analyses were adjusted for age and TNM stage. Subgroup sizes are indicated in each panel. For each comparison, the hazard ratio (HR), 95% confidence interval (CI), and p-value (log-rank test) are shown.

PD-L1; Programmed cell death ligand 1, COX-2; Cyclooxygenase 2, CD; Cluster of differentiation, CD; Cluster of differentiation, 15-PGDH; 15-hydroxy prostaglandin dehydrogenase.

#### Supplementary Fig. 8. Celecoxib treatment reduces PD-L1 expression in colon cancer (CC) cells via COX-2 inhibition.

Confocal immunofluorescence analysis of PD-L1 and COX-2 expression in colon cancer (CC) cell lines. **a,** RKO (MSI-H/dMMR) and **b,** SW480 (MSS/pMMR) cells were treated with IFN-γ (50 ng/mL, 24 h), followed by COX-2 inhibitor (Celecoxib) (10 µM, 24 h), prior to fixation and staining. Representative confocal images show co-expression of COX-2 and PD-L1, with insets (white dotted lines) highlighting regions of interest (ROI). **a’, b’,** Quantification of PD-L1 mean fluorescence intensity (MFI) was performed using ImageJ macros and presented as box plots (with medians and 10^th^ to 90^th^ percentiles), and the filled circles indicate the MFI in the top and bottom deciles; n = 3 replicates, >90 cells per group. Scale bar, 10 µm. Asterisks indicate statistical significance (mean ± S.E.M., two-tailed Student’s t-test, ***p ˂ 0.001).

#### Supplementary Fig. 9. Risk prediction models integrating immune and inflammatory features.

**a,** ROC curve comparing AUCs for composite risk scores using TS⁺ PD-L1 + COX-2 + CD4 (AUC = 0.712) and TS⁺ PD-L1 + COX-2 + 15-PGDH (AUC = 0.637) in the internal cohort. **b-c,** ROC comparisons from GSE39582 cohort for **b,** *CD274* + *CD8A* and **c,** *CD274* + *PTGS2* + *CD8A*, with or without clinical variables (age, sex, TNM stages).

PD-L1, programmed cell death ligand 1; COX-2, cyclooxygenase-2; CD, cluster of differentiation; 15-PGDH, 15-hydroxyprostaglandin dehydrogenase; TS, tumor stroma; AUC, area under the curve.

**Supplementary Fig. 10. Immunohistochemistry-based assessment of mismatch repair (MMR) proteins in colorectal cancer (CRC) tissue microarrays.**

**a,** Representative immunohistochemistry images (10x and 40x magnification) of tissue microarray (TMA) core showing nuclear staining of MSH2, MSH6, MLH1, and PMS2 expression used to evaluate the mismatch repair (MMR) status of the patients in the internal cohort based on the H-score quantification using Halo Imaging software. Patients with preserved nuclear staining for all four markers in the tumor cells were considered as MMR-proficient (pMMR) or microsatellite stable (MSS), and patients with loss of nuclear staining in either of the four markers were categorized as MMR-deficient (dMMR) or microsatellite instable (MSI). Insets (dotted boxes) indicate zoomed regions of interest. Expression of each marker was quantified using the Halo imaging system and presented as an H-score.

#### References:

[1] T. Li, J. Fu, Z. Zeng, D. Cohen, J. Li, Q. Chen, B. Li, and X.S. Liu, TIMER2.0 for analysis of tumor-infiltrating immune cells. Nucleic Acids Research 48 (2020) W509-W514.
