# Supplementary material for: Integrating COX-2, stromal PD-L1, and T-cell infiltration enhances prognostic stratification in colorectal cancer
Source: BMC Cancer. 2025 Sep 16;25:1424. doi: 10.1186/s12885-025-14927-x (PMC12442288; doi:10.1186/s12885-025-14927-x)
Supplement: Supplementary file 3 — Supplementary Material 3. [file 12885_2025_14927_MOESM3_ESM.docx]

| Clinicopathologic variable | GSE39582 |
| --- | --- |
|  | Overall Survival  **HR 95% CI p-value** |
| Age  ≥65 vs. ˂65 | 1.703 1.174 – 2.469 0.005 |
| Gender  Male vs. Female | 1.378 0.993 – 1.913 0.055 |
| TNM Stage  All stages | 0.195 0.023 – 1.682 0.223 |
| *CD274* (PD-L1) expression  Categorical (High vs. Low) | 1.128 0.819 – 1.553 0.460 |
| *PTGS2* (COX-2) expression  Categorical (High vs. Low) | 0.658 0.438 – 0.987 0.043 |
| *CD8A* (CD8) expression  Categorical (High vs. Low) | 0.746 0.541 – 1.028 0.073 |
| Microsatellite Instability (MSI)  MSS vs MSI | 0.943 0.445 – 1.999 0.917 |

**Supplementary Table 2:** Survival and associations with clinicopathologic factors in the external dataset GSE39582 using Cox regression.
