# Supplementary figures and images for: Integrating COX-2, stromal PD-L1, and T-cell infiltration enhances prognostic stratification in colorectal cancer
Source: BMC Cancer. 2025 Sep 16;25:1424. doi: 10.1186/s12885-025-14927-x (PMC12442288; doi:10.1186/s12885-025-14927-x)

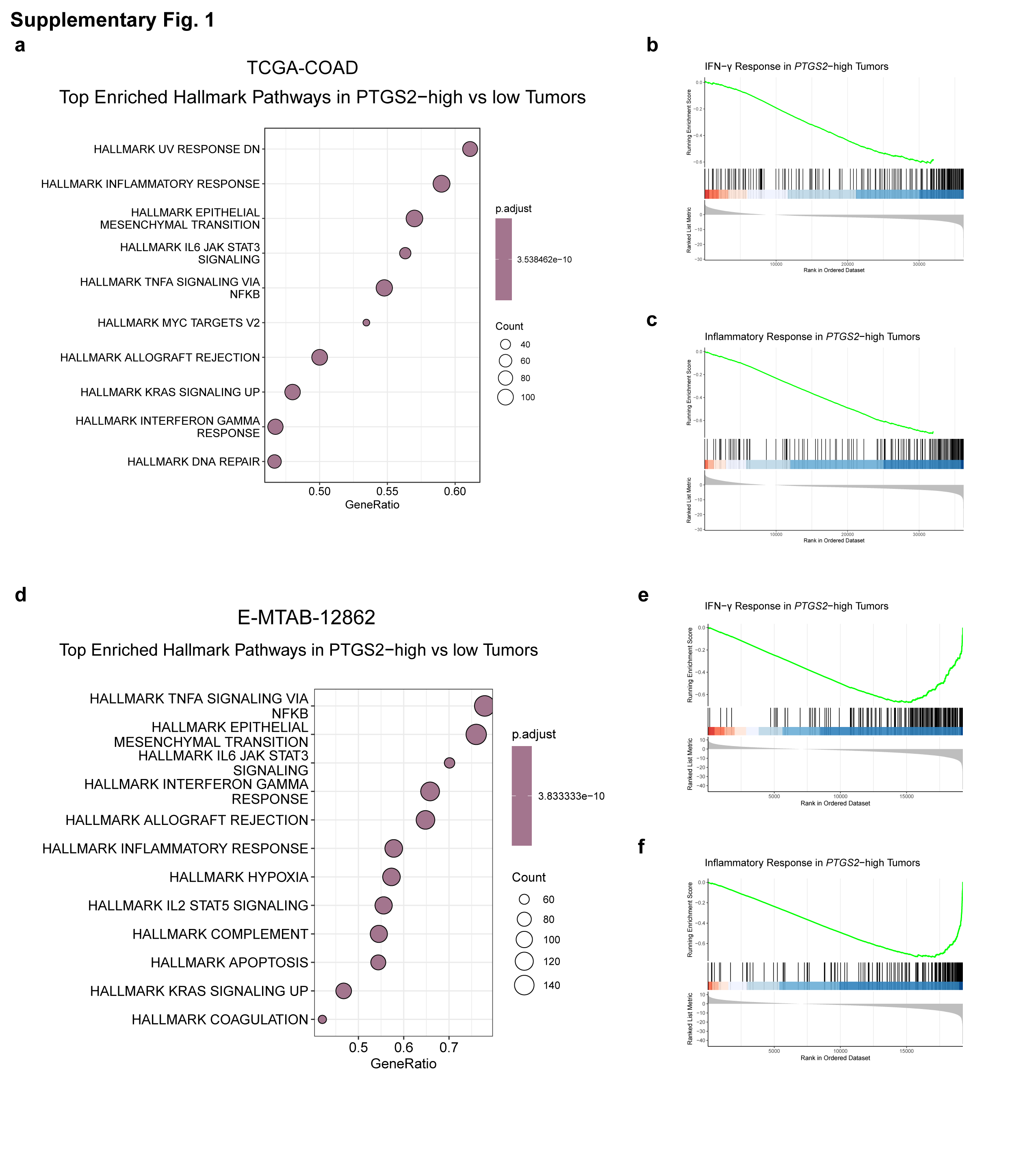

Supplement: Supplementary file 4 — Supplementary Material 4. [file 12885_2025_14927_MOESM4_ESM.tif]

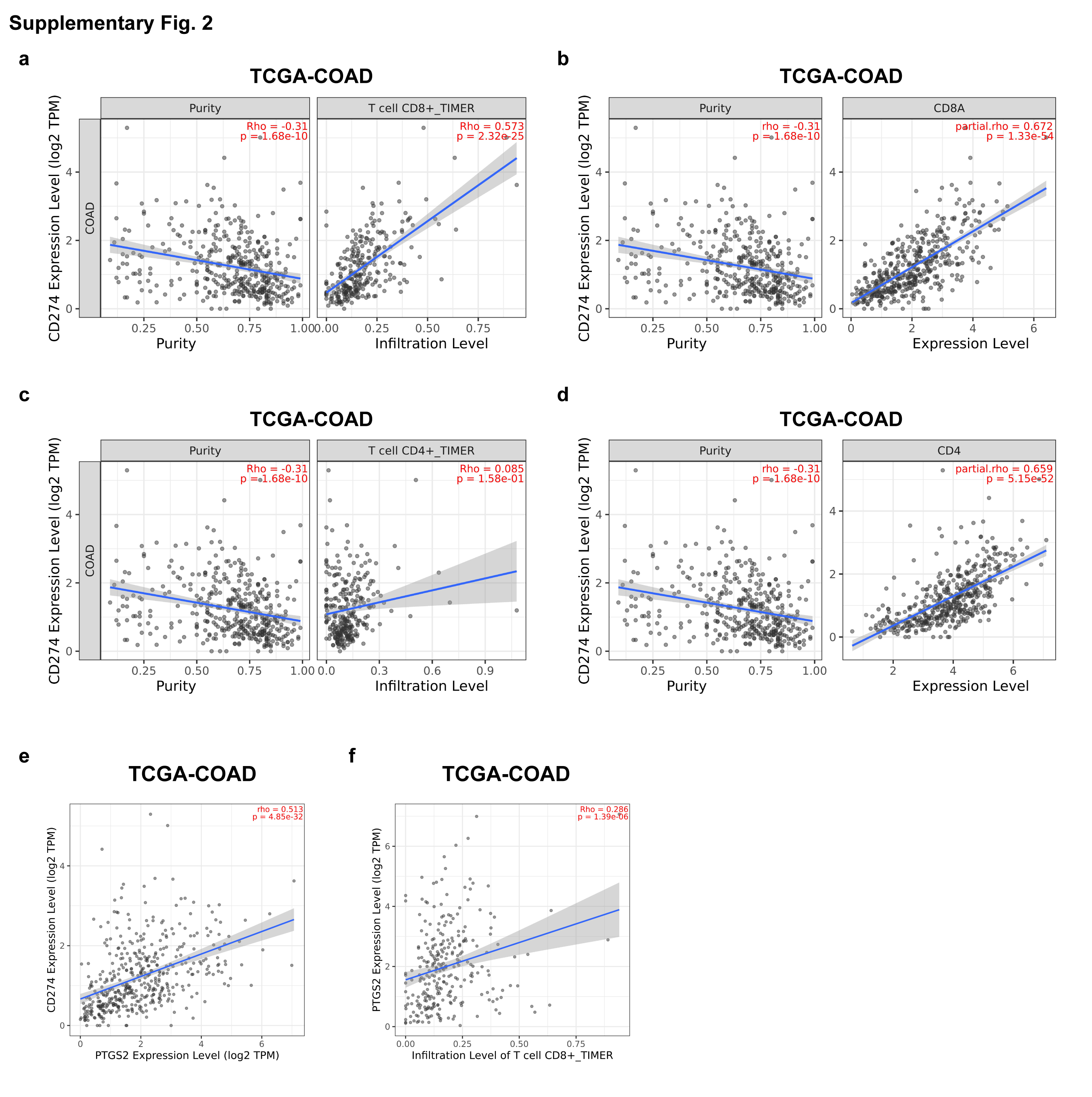

Supplement: Supplementary file 5 — Supplementary Material 5. [file 12885_2025_14927_MOESM5_ESM.tif]

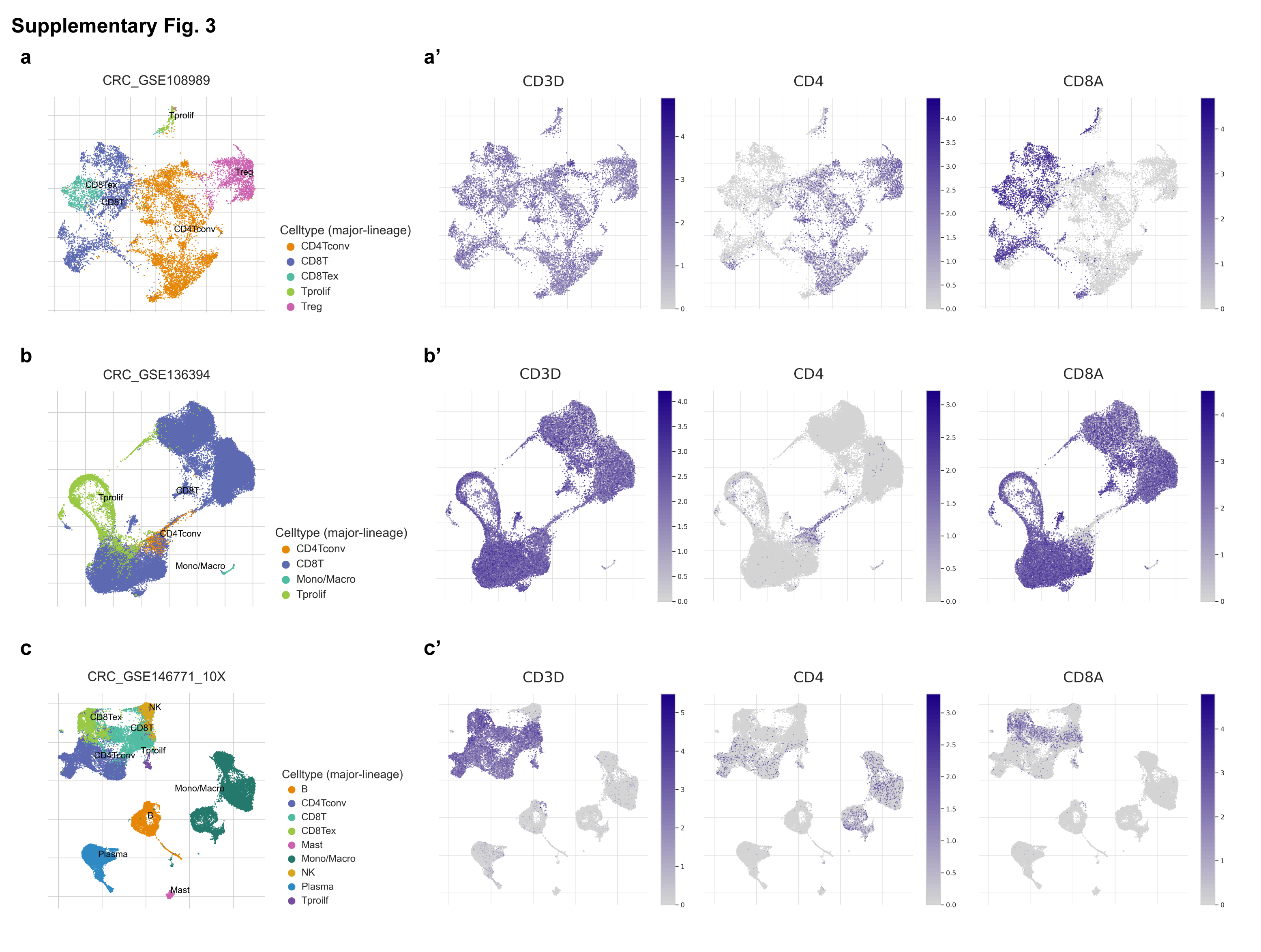

Supplement: Supplementary file 6 — Supplementary Material 6. [file 12885_2025_14927_MOESM6_ESM.tif]

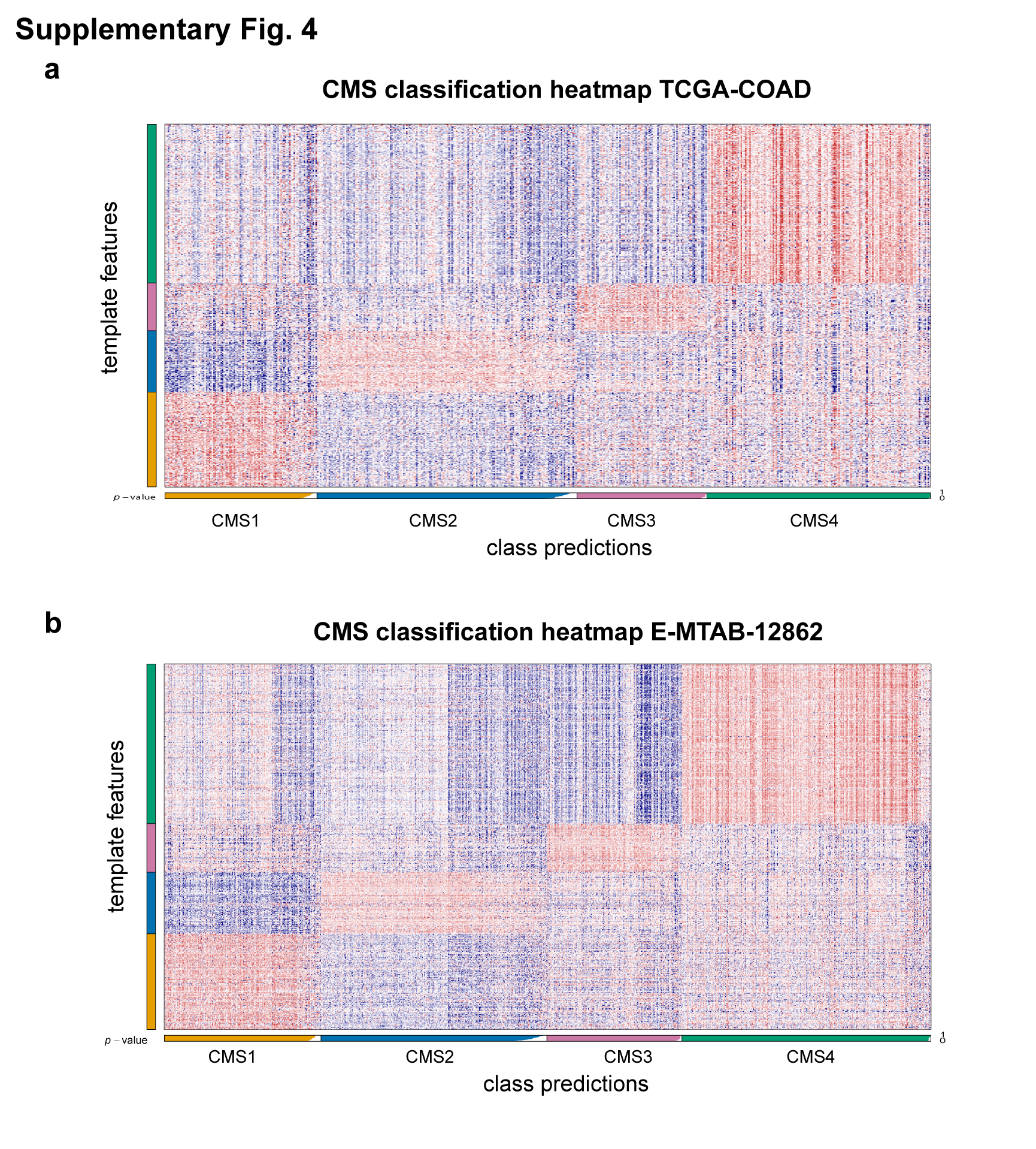

Supplement: Supplementary file 7 — Supplementary Material 7. [file 12885_2025_14927_MOESM7_ESM.tif]

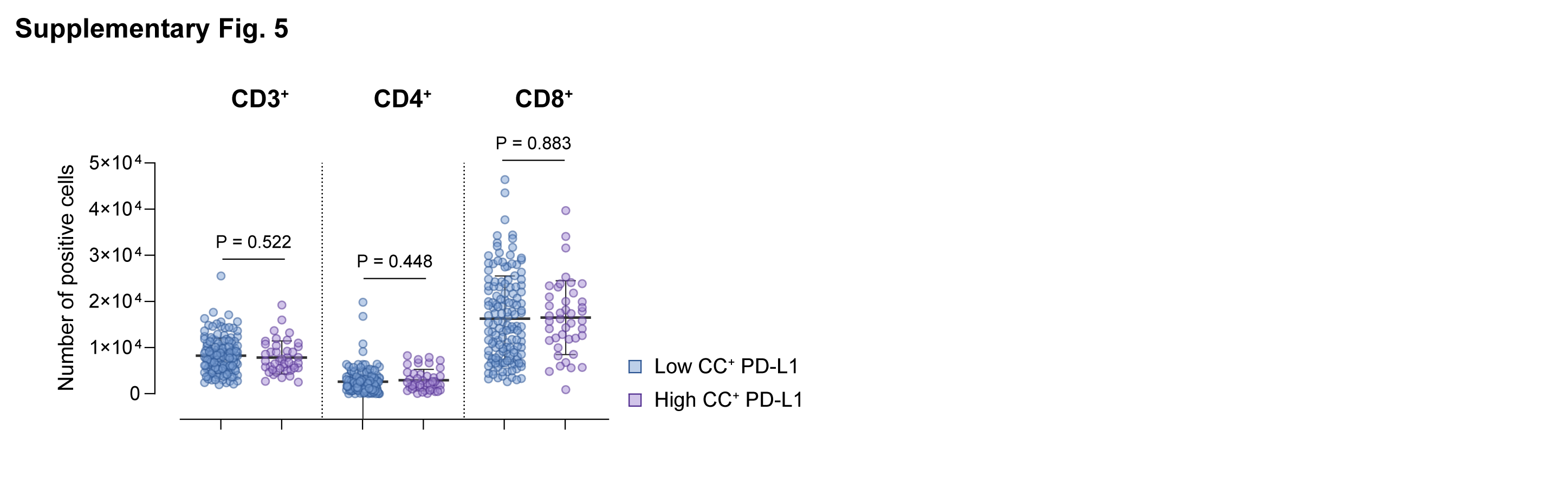

Supplement: Supplementary file 8 — Supplementary Material 8. [file 12885_2025_14927_MOESM8_ESM.tif]

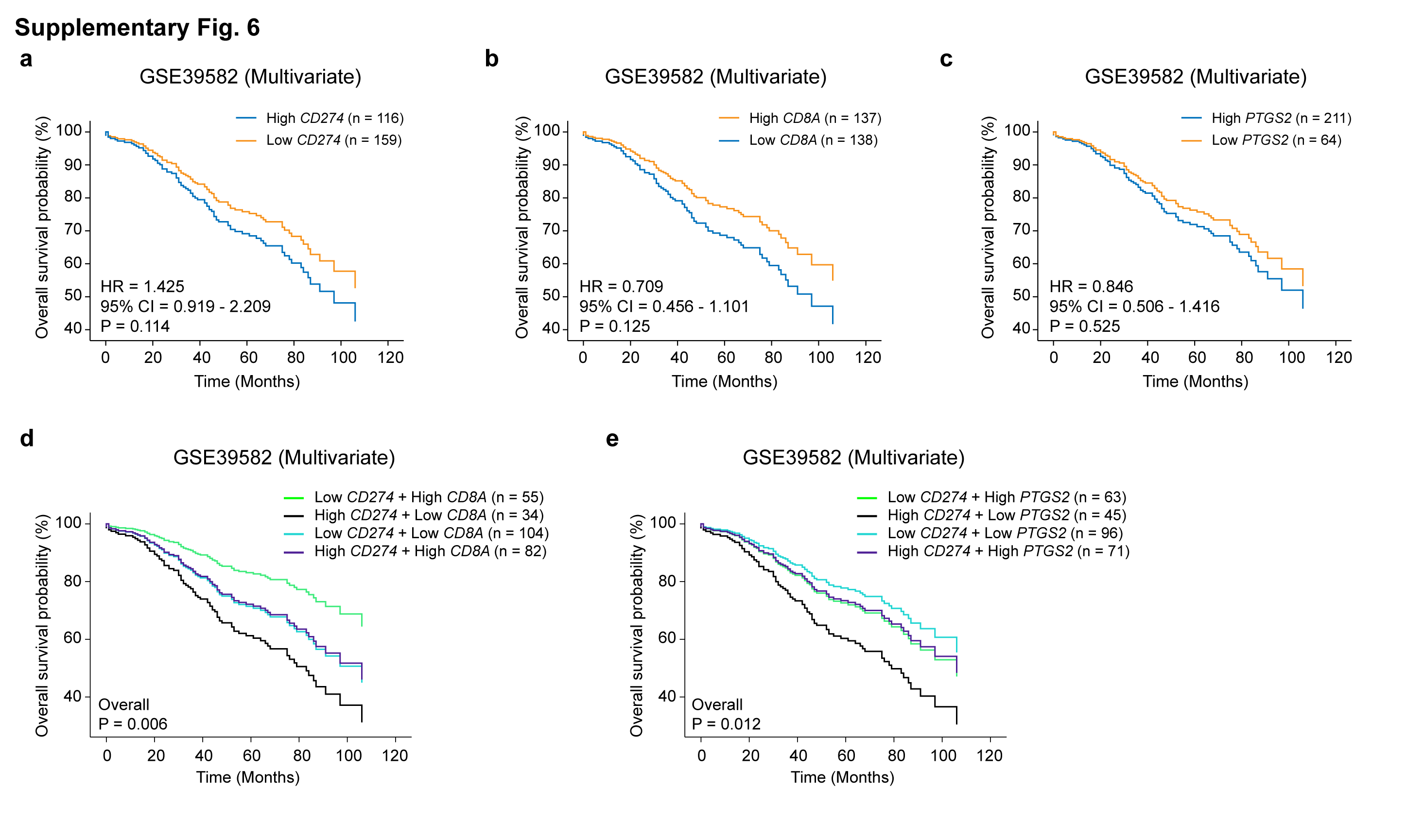

Supplement: Supplementary file 9 — Supplementary Material 9. [file 12885_2025_14927_MOESM9_ESM.tif]

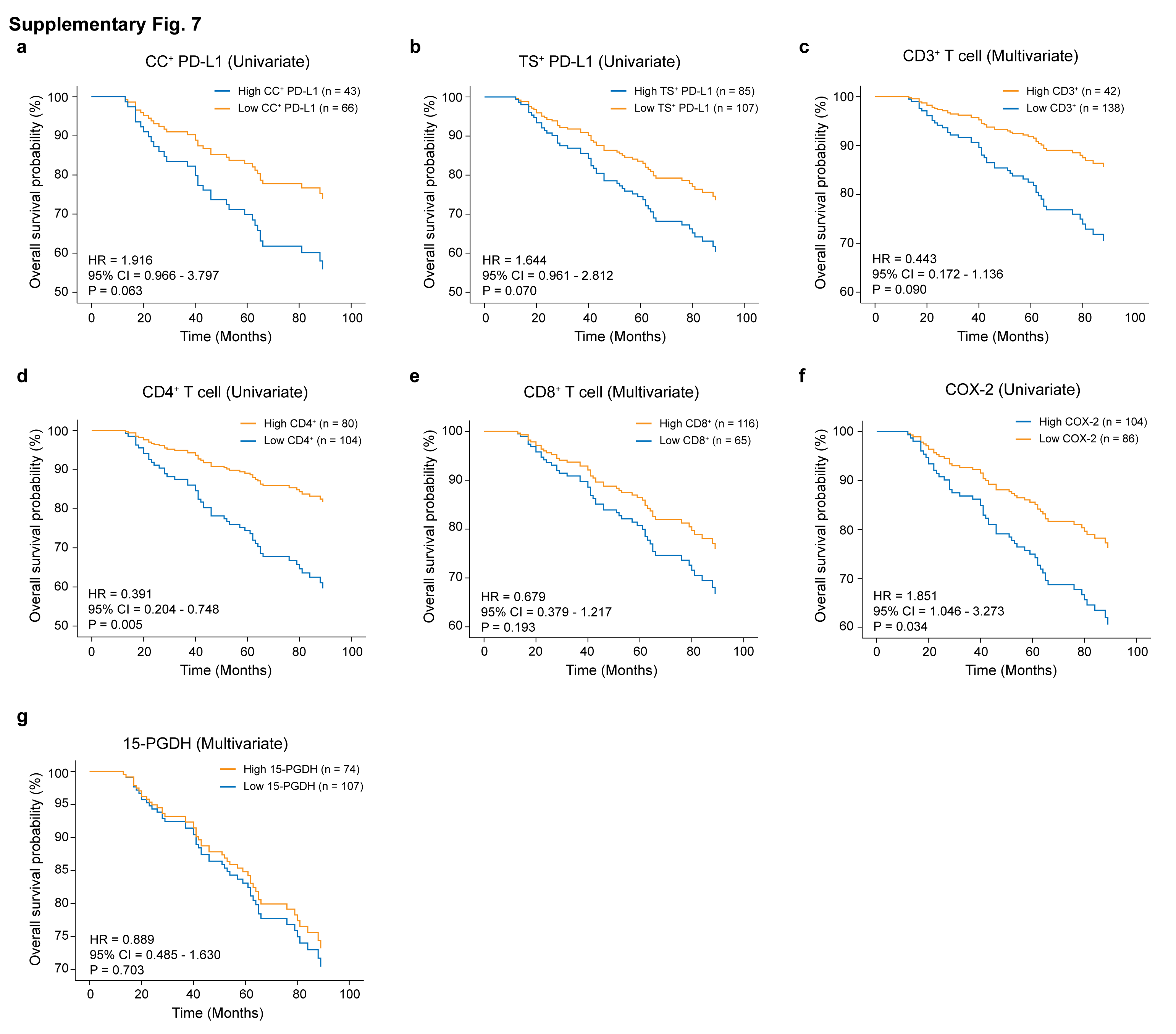

Supplement: Supplementary file 10 — Supplementary Material 10. [file 12885_2025_14927_MOESM10_ESM.tif]

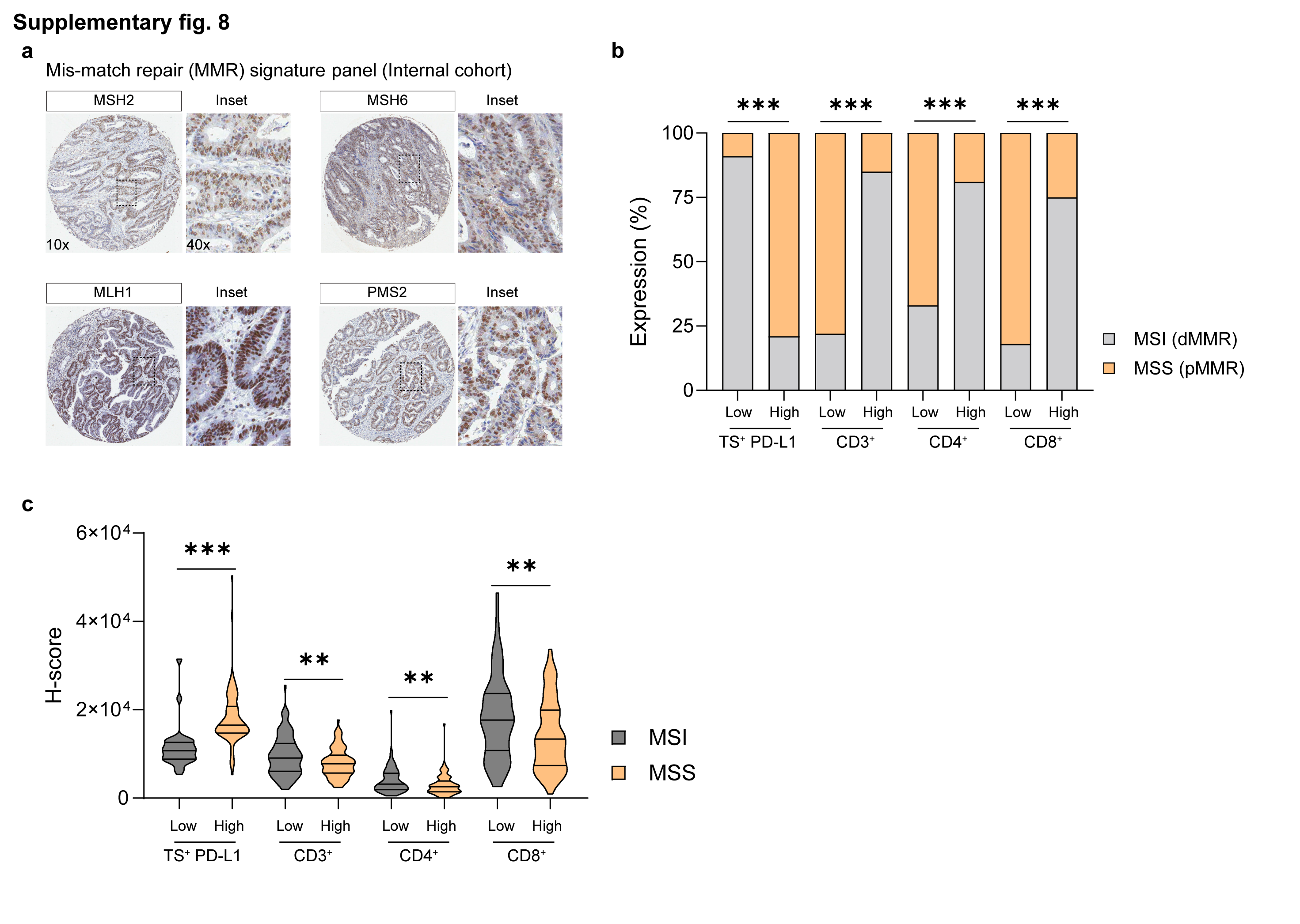

Supplement: Supplementary file 11 — Supplementary Material 11. [file 12885_2025_14927_MOESM11_ESM.tif]

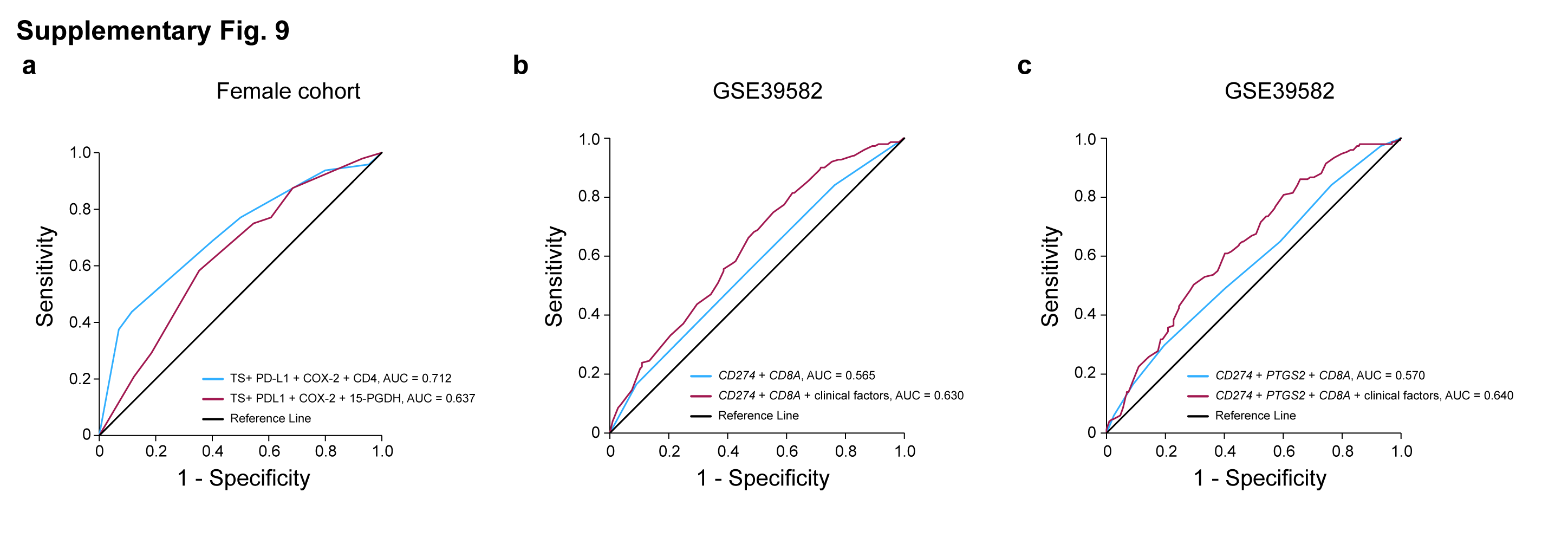

Supplement: Supplementary file 12 — Supplementary Material 12. [file 12885_2025_14927_MOESM12_ESM.tif]

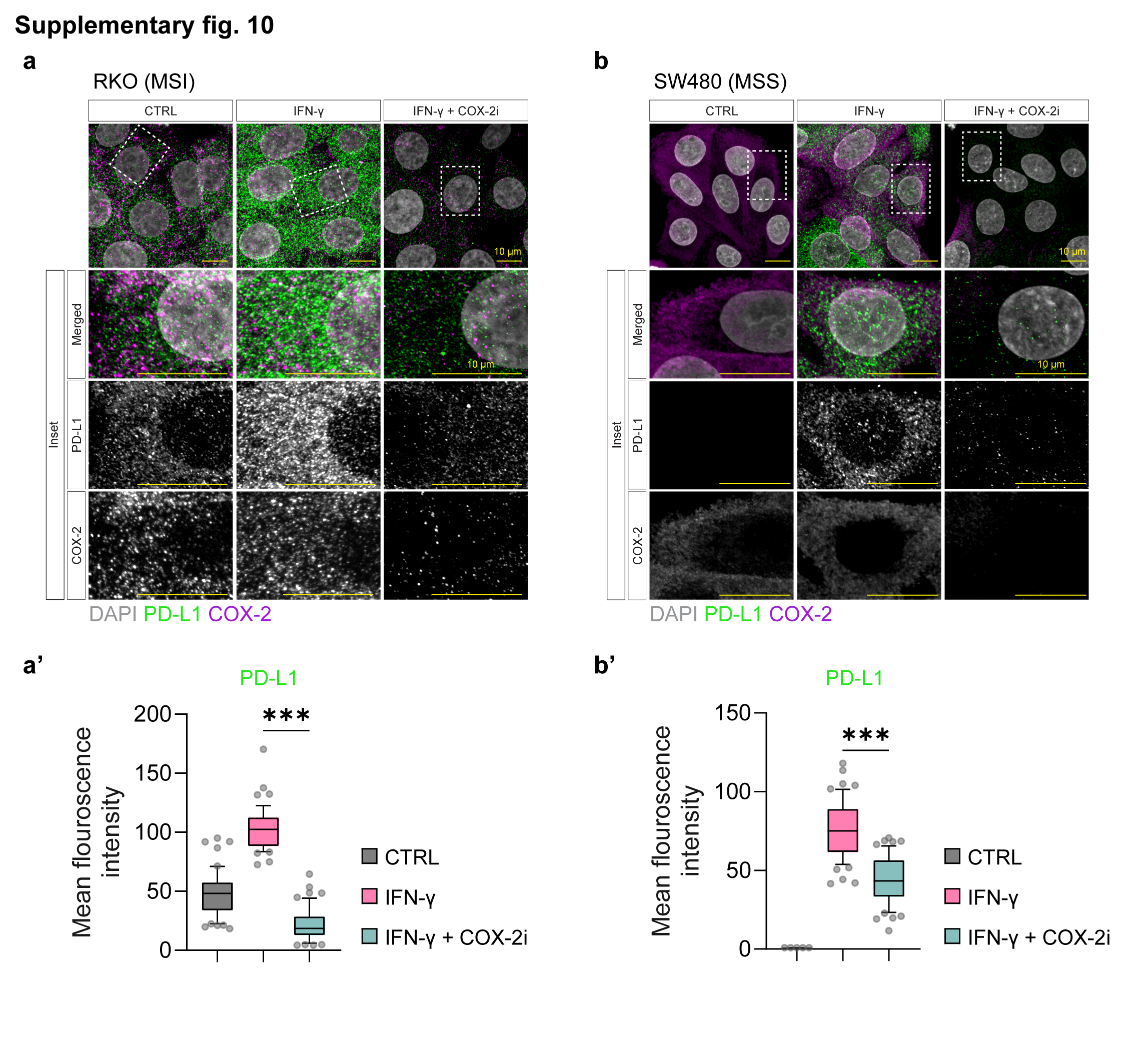

Supplement: Supplementary file 13 — Supplementary Material 13. [file 12885_2025_14927_MOESM13_ESM.tif]
